# Light-regulated microRNAs shape dynamic gene expression in the zebrafish circadian clock

**DOI:** 10.1371/journal.pgen.1011545

**Published:** 2025-01-08

**Authors:** Zuo Wang, Shuang Wang, Yi Bi, Alessandra Boiti, Shengxiang Zhang, Daniela Vallone, Xianyong Lan, Nicholas S. Foulkes, Haiyu Zhao

**Affiliations:** 1 School of Life Sciences, Gansu Key Laboratory of Biomonitoring and Bioremediation for Environmental Pollution, Lanzhou University, Lanzhou, China; 2 Institute of Biological and Chemical Systems, Biological Information Processing (IBCS-BIP), Karlsruhe Institute of Technology (KIT), Eggenstein-Leopoldshafen, Germany; 3 Key Laboratory of Animal Genetics, Breeding and Reproduction of Shaanxi Province, College of Animal Science and Technology, Northwest A&F University, Yangling, China; Tel-Aviv University Department of Biochemistry and Molecular Biology, ISRAEL

## Abstract

A key property of the circadian clock is that it is reset by light to remain synchronized with the day-night cycle. An attractive model to explore light input to the circadian clock in vertebrates is the zebrafish. Circadian clocks in zebrafish peripheral tissues and even zebrafish-derived cell lines are entrainable by direct light exposure thus providing unique insight into the function and evolution of light regulatory pathways. Our previous work has revealed that light-induced gene transcription is a key step in the entrainment of the circadian clock as well as enabling the more general adaptation of zebrafish cells to sunlight exposure. However, considerable evidence points to post-transcriptional regulatory mechanisms, notably microRNAs (miRNAs), playing an essential role in shaping dynamic changes in mRNA levels. Therefore, does light directly impact the function of miRNAs? Are there light-regulated miRNAs and if so, which classes of mRNA do they target? To address these questions, we performed a complete sequencing analysis of light-induced changes in the zebrafish transcriptome, encompassing small non-coding RNAs as well as mRNAs. Importantly, we identified sets of light-regulated miRNAs, with many regulatory targets representing light-inducible mRNAs including circadian clock genes and genes involved in redox homeostasis. We subsequently focused on the light-responsive miR-204-3-3p and miR-430a-3p which are predicted to regulate the expression of cryptochrome genes (*cry1a* and *cry1b*). Luciferase reporter assays validated the target binding of miR-204-3-3p and miR-430a-3p to the 3′UTRs of *cry1a* and *cry1b*, respectively. Furthermore, treatment with mimics and inhibitors of these two miRNAs significantly affected the dynamic expression of their target genes but also other core clock components (*clock1a*, *bmal1b*, *per1b*, *per2*, *per3*), as well as the rhythmic locomotor activity of zebrafish larvae. Thus, our identification of light-responsive miRNAs reveals new intricacy in the multi-level regulation of the circadian clockwork by light.

## Author summary

Our study explored how light affects the biological clock of zebrafish, with a particular focus on the role of miRNAs, small molecules that regulate gene expression. Zebrafish provide a unique model for this research because their internal clocks can be directly reset by light. Through RNA-sequencing, we identified 1365 genes and 66 miRNAs that respond to light exposure, and constructed light-responsive mRNA-miRNA interaction networks, highlighting their roles in various biological processes including circadian clock regulation. We particularly focused on two miRNAs, miR-204-3-3p and miR-430a-3p, which can target core clock genes *cry1a* and *cry1b*, respectively and significantly impact the rhythmic gene expression and behavioral patterns of zebrafish larvae. This study not only advances our understanding of the mechanisms governing biological rhythms in zebrafish, but also provides new insight that could be relevant to other organisms, including humans. Ultimately, this work may contribute to developing better strategies for managing circadian rhythm disorders and improving overall health.

## 1. Introduction

Most organisms possess circadian clocks, the ubiquitous endogenous timing systems which optimally adapt and respond to 24h day-night cycle [1]. At a system level, the vertebrate circadian clock can be divided into master and peripheral clocks. The specialized master clock, also known as the "central pacemaker" serves to synchronize clocks in peripheral tissues and organs through various humoral pathways [2–7]. At the molecular level, the circadian clock is based on the interplay of transcriptional-translational feedback loops (TTFLs) that generate rhythmic oscillations in the expression of clock genes and their encoded proteins [8]. The positive limb of the core loop involves the transcription factors BMAL and CLOCK that heterodimerize and activate transcription of their target genes including *cryptochrome (cry)* and *period (per)* via binding to E-box enhancer elements, while the negative limb is represented by PER:CRY heterocomplexes that translocate into the nucleus and repress their own transcription by inhibiting CLOCK:BMAL-driven activation [9,10]. Additional TTFLs serve to stabilize this core loop and ensure its completion in circa 24 hours. Importantly, the intrinsic period length of circadian clock rhythmicity deviates from 24h and therefore requires daily synchronization by environmental time-cues, predominantly light [11]. While light-entrainment is known to be mediated by light-responsive gene expression, the detailed mechanisms underlying this process remain incompletely understood.

As a typical diurnal animal, the zebrafish serves as an attractive model for deciphering the vertebrate circadian clock mechanism [12]. Its pineal gland incorporates all the elements required for light-entrainment and the generation of circadian rhythms and thus is considered as a master clock organ [13]. Furthermore, in contrast to mammalian models, most zebrafish tissues and even zebrafish-derived cell lines possess peripheral circadian clocks which can be entrained by direct light exposure [14]. Thus, both its embryos and cell lines represent powerful models for investigating the molecular basis of the vertebrate circadian clock and its entrainment by light. According to previous studies, light-responsive gene expression plays crucial roles in transferring photic information to the circadian clock machinery. For example, light induces the transcription of *cry1a* and *per2* in most tissues and cells, a process which has been demonstrated to underlie the entrainment of circadian clocks [15,16]. Systematic promoter analyses of circadian clock genes as well as light-inducible DNA repair genes have identified the D-box as a key cis-acting element in the regulation of light-responsive gene transcription [17–19]. Transcriptome analyses of zebrafish embryos, larvae, cell cultures, pineal glands and other tissues have revealed light regulation of various biological processes including DNA repair function in addition to the control of circadian rhythmicity [20–22]. All these studies have contributed to a general understanding of the mechanisms whereby light entrains circadian clocks at the transcriptional level.

Accumulating evidence suggests that clock genes are the target of modulation by many other mechanisms including miRNA-mediated post-transcriptional regulation [23]. MicroRNAs (miRNAs) are a class of evolutionarily conserved small non-coding RNAs, 18–22 nucleotides in length. They can bind to complementary target sites within the 3′UTRs of specific mRNAs and down-regulate their expression through mRNA degradation or translational repression [24]. Recently, miRNAs have been implicated in the circadian clock machinery as post-transcriptional regulators [22,25–27]. For example, miRNAs including let-7, miR-204, miR-210, miR-263b, miR-279 and miR-24 have been shown to regulate the expression of clock genes and thereby fine-tune the 24-hour circadian rhythmicity [28–34]. However, the precise functional contribution of miRNAs to the entrainment of the circadian clock mechanism by light remains unclear.

In this study, we aim to decipher the role of miRNAs in light-dependent gene regulatory networks in vertebrates. Based on sequencing of the light-responsive transcriptome in zebrafish larvae, we employed bioinformatic integration of light-regulated mRNA and miRNA profiles to predict an mRNA-miRNA interaction network. We then performed functional analyses of two light-regulated miRNAs, miR-204-3-3p and miR-430a-3p, and revealed that these miRNAs serve to control the expression of light- as well as clock-regulated core circadian clock genes. Furthermore, modulation of miR-204-3-3p and miR-430a-3p levels by specific miRNA mimics and inhibitors affected the circadian profile of rhythmic locomotor activity in zebrafish larvae. Thus, our study reveals the presence of an intricate, multi-level regulation of clock gene expression which involves light-regulated miRNAs in the directly light-entrainable zebrafish clock.

## 2. Materials and methods

### 2.1 Ethics statement and zebrafish maintenance

Zebrafish husbandry and experimental procedures were reviewed and approved by the Animal Care and Ethics Committee of the School of Life Sciences at Lanzhou University (Approval No. EAF2021022) and were performed in accordance with the *Guide for the Care and Use of Laboratory Animals*. Every effort was made to minimize animal suffering. Adult zebrafish (*Danio rerio*, AB strain) were maintained in an automated water-cycling system (Thmorgan, China) at 28°C under 14h:10h light: dark conditions, and were fed twice daily with freshly hatched brine shrimps, as previously described [19,35].

### 2.2 Sampling of zebrafish larvae for RNA-sequencing

Zebrafish embryos were obtained from four pairs of adult males and females segregated in mating tanks overnight. Mating and spawning were induced by light exposure early the following morning. Fertilized embryos were collected and washed with standard E3 medium within one hour of laying. 2 hpf (hours post-fertilization) embryos were examined under a stereomicroscope, and normally developing embryos were selected. Aliquots of 40 embryos were transferred into fresh E3 medium in 10-cm petri dishes. The embryos were incubated in constant darkness (DD) at 28°C for 5 days in a programmable, thermostatically controlled incubator, followed by illumination with a fluorescent lamp (white light, intensity 1,000 lux) for 1h, 3h and 6h. To economize on the scale of sequencing as well as to minimize the possibility of developmental changes influencing the comparison of the 1h and 6h light treated larvae with DD controls, the DD control group was sampled at 3h, in the middle of the 6h sampling period.

### 2.3 Construction of RNA libraries and sequencing

Total RNA was isolated and purified from zebrafish larvae using Trizol reagent (Invitrogen, USA) according to the manufacturer’s instructions. The quantity and quality of the RNA samples were assessed using a NanoDrop ND-1000 and an Agilent 2100 bioanalyzer. Only high quality samples were used for downstream library construction and subsequent mRNA and miRNA sequencing, performed as previously described **(S1 Fig)** [36,37]. For mRNA sequencing, ribosomal RNA was depleted using the Ribo-Zero rRNA Removal Kit (Illumina, USA), and then the cleaved RNA fragments were reverse transcribed to create sequencing libraries. For miRNA sequencing, miRNAs were reverse transcribed using TruSeq Small RNA Sample Prep Kits (Illumina, USA) to generate sequencing libraries. Index codes were assigned to the samples, which were sequenced on an Illumina NovaSeq6000 platform with 125 bp paired-end and 50 bp single-end reads, by LIANCHUAN Biotechnology Co., Ltd (Hangzhou, China).

### 2.4 Bioinformatic analyses

Raw sequencing data were firstly pre-processed, which included background correction and data normalization. Differentially expressed (DE)-mRNAs were identified with fold change |FC| > 1.5 and p value < 0.05, while DE-miRNAs were defined with fold change |FC| > 1.2 and p value < 0.1. Heat maps of DE-mRNAs and DE-miRNAs were plotted using the R package *ggplot2*. **Clustering Analysis:** to define the expression patterns of light-responsive mRNAs, hierarchical clustering based on Pearson correlation was performed using the MEV program (vision 4.9.0) [38]. The results were visualized as a heat map. **Prediction of miRNA Target Genes:** miRNA target gene prediction was conducted using miRanda (vision 1.9) and Targetscan (vision 6.2) with default parameters. The miRanda score threshold was set at energy < -20 kcal/mol, and the Targetscan percentile was set at score > 70. Overlapping target genes predicted by both programs were considered potential targets of DE-miRNA, and the mRNA-miRNA interaction network was visualized using Cytoscape software (vision 3.8.2). **Functional Annotations:** to explore the potential roles of light-responsive mRNAs and miRNA target genes, Gene Ontology (GO) and Kyoto Encyclopedia of Genes and Genomes (KEGG) pathway enrichment analyses were performed and visualized using *ggplot2*. **Protein-Protein Interaction (PPI) Analyses:** functional interactions between the protein products of light-responsive genes were investigated using the STRING database in Cytoscape. To identify hub genes within the interaction networks, the top 10 genes with the highest maximal clique centrality (MCC) values were identified and displayed using the cytoHubba plugin in Cytoscape [39]. **RNA Secondary Structure Prediction:** the secondary structures of miRNA precursors were predicted by obtaining pre-miRNA sequences from the miRBase database (http://www.mirbase.org/) and submitting them to RNAFold, using recommended parameters [40–42]. **Comparative Sequence Analysis:** pre-miRNA sequences from zebrafish (*Danio rerio*), human (*Homo sapiens*), mouse (*Mus musculus*), African clawed frog (*Xenopus leavis*), Carolina anole (*Anolis carolinensis*), Japanese medaka (*Oryzias latipes*) and sea Lamprey (*Petromyzon marinus*) were obtained from the miRBase database and aligned using the MEGA software (version 7.0) [43].

### 2.5 Validation of light-responsive expression of mRNAs & miRNAs by qRT-PCR

Total RNA was extracted from zebrafish larvae using the TRIzol Reagent as previously described [17,19]. mRNA was reverse transcribed into cDNA using the PrimeScript RT Reagent Kit (CWBIO, China), while miRNA was reverse transcribed using the All-in-One miRNA First-Strand cDNA Synthesis Kit (Genecopoeia, USA). Quantitative real-time PCR (qRT-PCR) analyses were performed using the SYBR Green Kit (CWBIO, China) with three biological replicates per group. Relative expression levels of each gene and miRNA were calculated using the 2^-ΔΔCt^ method. *β-actin* (for mRNA) and small nuclear *U6* (for miRNA) were used as reference genes for data normalization. All primer sequences are provided in **S1 Table**.

### 2.6 Microinjection of miRNA mimics/inhibitors

miRNA mimics are designed with sequences that are identical to endogenous mature miRNA molecules and thereby enable up-regulation of their activity [44,45]. Inhibitors are synthesized with sequences that are complementary to mature miRNA molecules and thereby specifically hybridize with them [46,47]. This prevents association with the RNA-induced silencing complex (RISC), which is crucial for the miRNA-mediated gene-silencing pathway [48,49]. Furthermore, miRNA inhibitors specifically prevent miRNAs from binding to their target mRNAs. Zebrafish embryos at the single or two-cell stages were microinjected with miRNA mimics or inhibitors (10 μM) or equivalent concentrations of a negative control (NC) using capillary needles and the WPI microinjection system (USA). After injection, the embryos were cultured at 28°C under 12-h:12-h light: dark cycles for subsequent experiments. The miRNA mimics & inhibitors used in this study were synthesized by Shanghai GenePharma (China). They are small, chemically synthesized and optimized nucleic acids, which possess 2’-O-methylation at the 3’ terminal ribose to increase the binding affinity to RNA molecules and enhance resistance to exonucleases. The sequences are listed in **S2 Table**.

### 2.7 Luciferase reporter assays

The 3′UTRs of zebrafish *cry1a* and *cry1b* were amplified and cloned into the psicheck-2 vector (Promega, USA) downstream of the luciferase gene, using Xho I and Not I restriction sites, thus generating *psicheck-2-cry1a-3′UTR* and *psicheck-2-cry1b-3′UTR*. The putative miR-204-3-3p and miR-430a-3p binding sites within the 3′UTRs were mutated using the QuickMutation Plus Kit (Beyotime, China), generating the mutated constructs *psicheck-2-cry1a-3′UTR MUT* and *psicheck-2-cry1b-3′UTR MUT*, respectively. 293T cells were cultured in 96-well plates and transfected with the luciferase plasmids along with miRNA mimics or a negative control (mimics-NC) using Lipofectamine 2000 (Invitrogen, USA) according to the experimental design. Cells were harvested 24h after transfection, and bioluminescence was measured using the Dual-Luciferase Assay System (Promega, USA) following the manufacturer’s recommendations. Primer sequences are provided in **S3 Table**.

### 2.8 Locomotor activity tests of zebrafish larvae

Zebrafish embryos microinjected with miRNA mimics, inhibitors, or controls were maintained at 28°C under 12h light: 12h dark cycles for 4 days. At 5 dpf, larvae without any morphological abnormalities were selected and placed individually in 48-well plates within the observation chamber of the DanioVision Tracking System (Noldus IT). The larvae were allowed to acclimate for at least 30 min before their locomotor activity was continuously recorded at 28°C under either 12h light: 12h dark cycles or 2 days of constant darkness. Various behavioral parameters including distance moved and periodic amplitudes were analyzed using EthoVision XT 15.0 (Noldus IT) and SPSS 23.0 software.

### 2.9 Data analysis

Statistical analyses were conducted using RStudio, GraphPad Prism 8, and SPSS 23.0. All numerical results are presented as mean ± SEM. Data subjected to statistical tests were firstly checked for normality and homoscedasticity. Significant differences were determined using Student’s t-test or analysis of variance (ANOVA) followed by multiple comparison post-test. P values < 0.05 were considered statistically significant, with p < 0.05, p < 0.01, p < 0.001 represented by *, ** and ***, respectively. Detailed statistical information is provided in **S4 Table**.

## 3. Results

### 3.1 Characterization of the light-responsive coding transcriptome in zebrafish larvae

**Fig 1:**
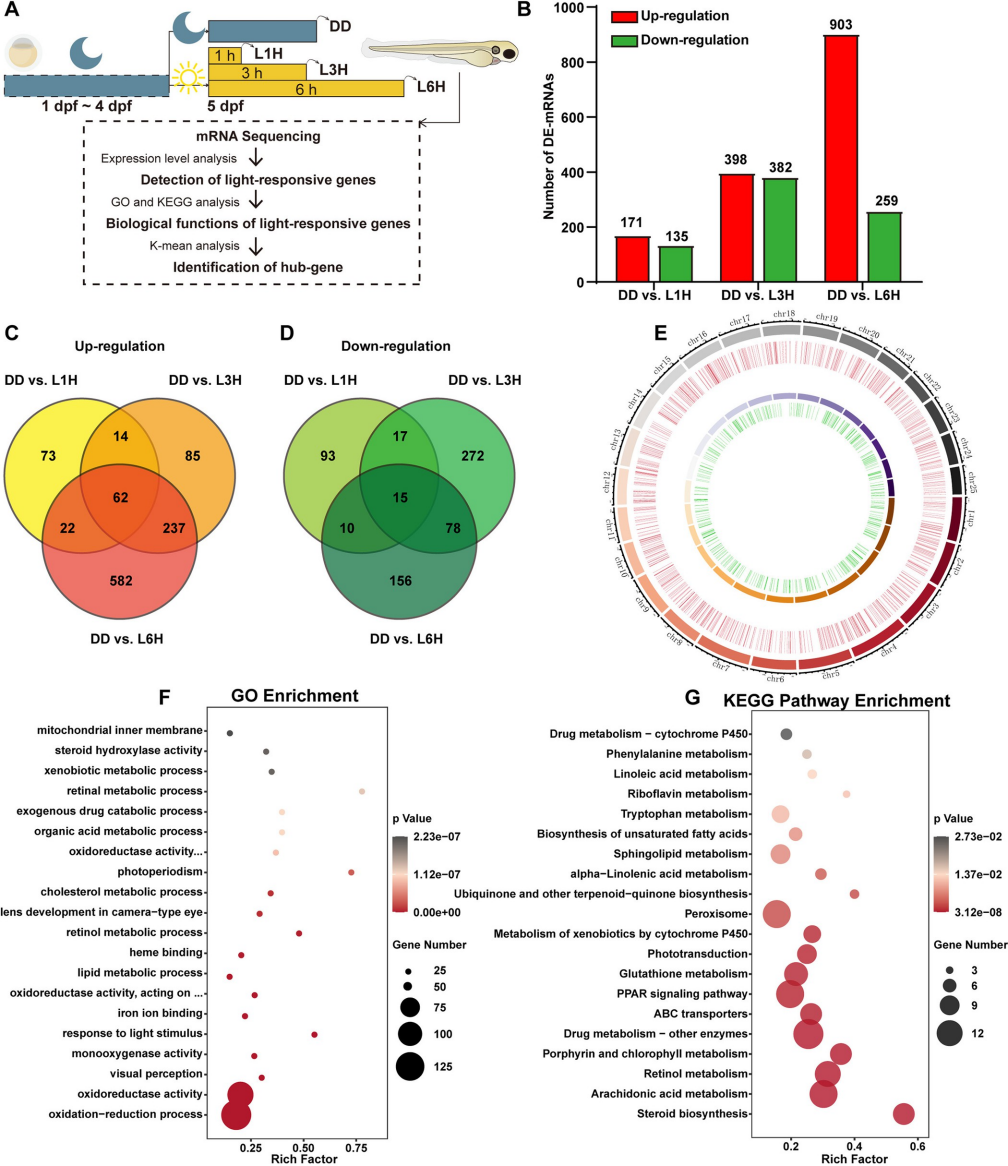
Overview of the light-responsive transcriptome in zebrafish larvae. **(A)** Schematic workflow illustrating the identification and expression profiling of light-responsive genes in zebrafish larvae. **(B-D)** Summary of differentially expressed genes (DEGs) identified in comparisons between larvae raised in constant darkness (DD) and those exposed to light for 1h (L1H), 3h (L3H), and 6h (L6H). Venn diagrams illustrate the overlap of up-regulated (C) and down-regulated (D) genes across the three comparisons. **(E)** Chromosomal distribution of light-responsive genes, visualized in a circular ideogram. **(F)** Gene Ontology (GO) analysis highlighting the top 20 enriched GO terms associated with light-responsive genes. **(G)** Kyoto Encyclopedia of Genes and Genomes (KEGG) pathway analysis showing the top 20 enriched pathways. Bubble size indicates the number of genes enriched in each pathway, with color intensity corresponding to the p-value.

Light-induced gene expression is a crucial step in the entrainment of the circadian clock as well as the more general adaptation of cells to sunlight in fish [12,50,51]. Considerable evidence points to post transcriptional regulatory mechanisms, notably miRNAs, playing an essential role in shaping dynamic changes in mRNA levels. Does light directly impact the function of miRNAs? To address this question, we performed a complete sequencing analysis of light-induced changes in the zebrafish transcriptome including small non-coding RNAs as well as mRNAs. Zebrafish embryos/larvae were raised in constant darkness (DD) for 5 days before being subjected to light pulses of 1, 3, or 6h. A single DD control group was sampled at 3h, in the middle of the 6h sampling period in order to economize on the scale of sequencing as well as to minimize the possibility of developmental changes influencing the comparison of the 1h and 6h light treated larvae with DD controls. Larval samples were pooled, followed by RNA extraction, sequencing and bioinformatics analysis **(Fig 1A)**. The Agilent 5400 and Nanodrop assay systems were used to verify sufficient yields of high-quality RNA for the construction of sequencing libraries **(S2 Fig and S5 Table)**. As a first step we initially focused on identifying light-regulated mRNAs and thereby defining potential functional targets for miRNA regulation. The summary of read counts for the sequencing data is presented in **S6 Table**. Briefly, 862.01–979.50 million of raw reads and 801.07–900.83 million of cleaned reads were generated in all our samples; the Q20 and Q30 scores exceeded 95% and GC contents were in the range of 62–67 %. Both the boxplot and density distribution diagram implied the uniform distribution of gene expression levels in different samples **(S3 Fig)**.

Differentially expressed mRNAs were identified based on thresholds of fold change |FC| > 1.5 and p-value < 0.05 for each pairwise comparison **(Fig 1B)**. Specifically, in comparison with the DD control group, 171 up- and 135 down-regulated genes were identified in the 1h light exposure group (L1H), 398 up- and 382 down-regulated genes were identified in the L3H group, 903 up- and 259 down-regulated genes were identified in the L6H group **(S7 Table)**. The number of light-responsive genes increased with prolonged light exposure. Next, Venn diagrams were plotted to show that a subset of genes were commonly up- or down- regulated across different light exposure durations **(Fig 1C and 1D)**. In addition, analysis of genome-wide distribution revealed that these light-responsive genes were dispersed across all 25 zebrafish chromosomes **(Fig 1E)**. Furthermore, Gene Ontology (GO) and Kyoto Encyclopedia of Genes and Genomes (KEGG) pathway enrichment analyses were carried out to annotate and categorize the biological functions and pathways affected by ambient light exposure in whole zebrafish larvae. GO analysis revealed significant enrichment in terms related to oxidation-reduction processes, oxidoreductase activity and visual perception, alongside other light-related terms such as response to light stimulus and photoperiodism **(Fig 1F)**. KEGG pathway analysis identified significant enrichment in biosynthetic and metabolic pathways, including steroid and unsaturated fatty acid biosynthetic, metabolism of arachidonic acid, retinol, drug and enzymes. Additionally, pathways including PPAR signaling, phototransduction and peroxisome function were significantly enriched in light-exposed zebrafish larvae **(Fig 1G)**. To validate our mRNA-sequencing data, we assessed the relative expression levels of a representative selection of light-responsive clock genes using qRT-PCR. Consistent with the sequencing results, the expression levels of clock genes such as *bmal1b*, *cry1a*, *cry1b*, *per2*, *per3*, along with DNA repair genes *ddb2*, *neil1* and *xpc* were significantly induced in light-exposed zebrafish larvae **(S4 Fig)**.

### 3.2 Clustering of light-responsive mRNAs and identification of Hub genes

**Fig 2:**
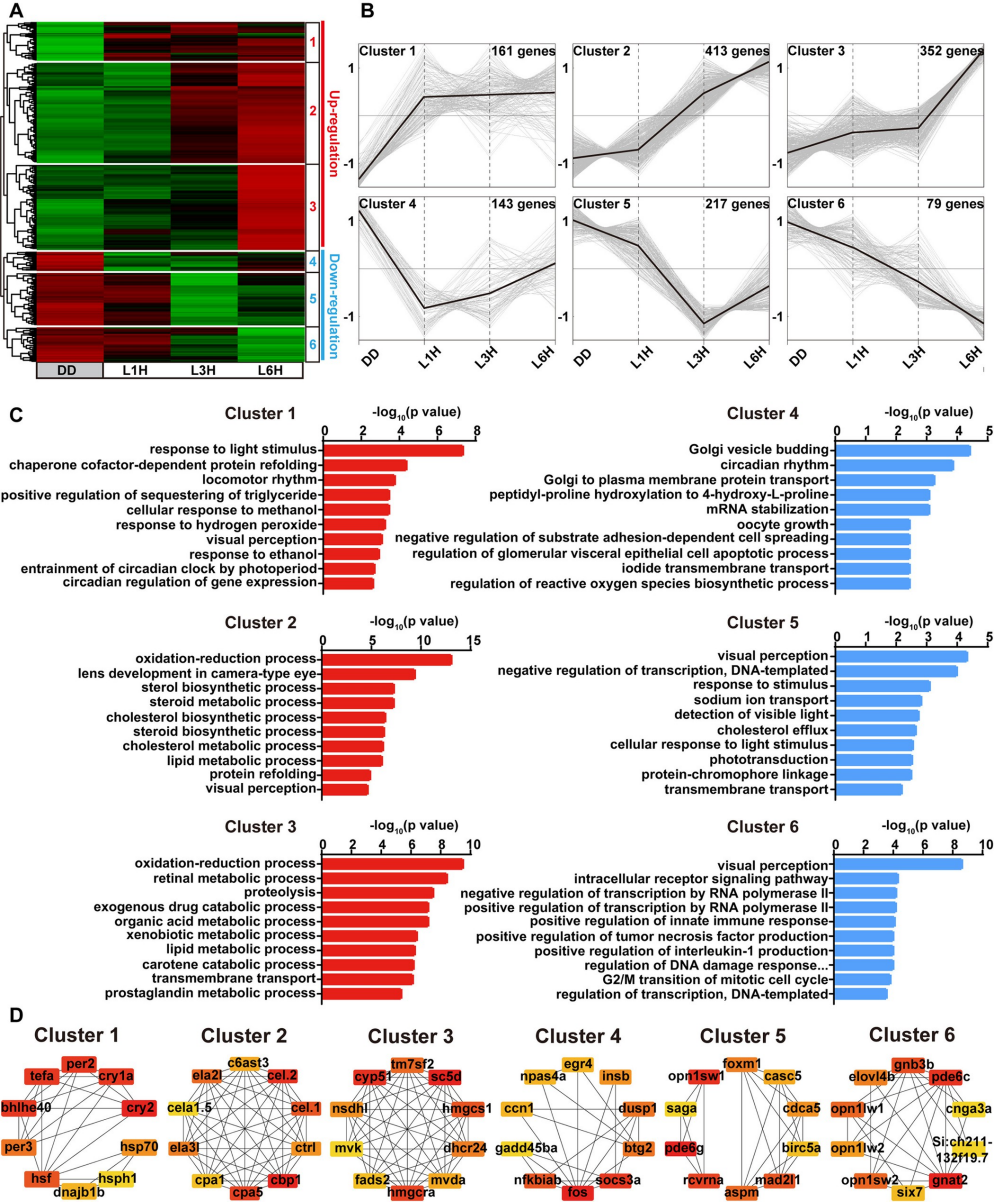
Cluster analysis, cluster-specific GO enrichment and protein-protein interaction networks of the zebrafish light-responsive mRNAs. **(A)** K-means clustering analysis of light-responsive mRNAs based on their expression patterns. The data is zero-centered to the entire dataset and displayed as a heatmap, with the color scale representing zero-centered signal intensity. **(B)** Expression patterns of DEGs from different clusters in response to light, with mean expression values for each cluster highlighted. **(C)** GO enrichment analysis (biological process) showing the top 10 representative terms for each cluster. **(D)** Protein-protein interaction networks within different clusters based on the STRING database. Primary subnetworks were identified using MCODE (Molecular Complex Detection) in Cytoscape, with the top 10 hub-genes for each cluster indicated.

To further dissect the differential expression profiles of the light-responsive mRNAs identified in zebrafish larvae, K-means clustering analysis was performed and these light-responsive mRNAs including 926 up-regulated and 439 down-regulated components can be further classified into six major clusters depending on their changes of expression patterns **(Fig 2A and 2B)**. Specifically, mRNAs in cluster 1 and 4 showed the most rapid response to ambient light, while those in cluster 3 and 6 exhibited relatively slower responses, and clusters 2 and 5 contained mRNAs with intermediate response profiles. Subsequently, GO analyses were conducted to determine the functional difference among different clusters **(Fig 2C)**. Interestingly, the fast-responding genes in clusters 1 and 4 were significantly enriched for GO terms related to the response to light stimulus, circadian rhythm, locomotor rhythm, entrainment of circadian clock by photoperiod, and circadian regulation of gene expression. In contrast, the light-induced genes in clusters 2 and 3 were highly enriched in oxidation-reduction process as well as a series of biosynthetic and metabolic processes. Meanwhile, the genes in cluster 5 and 6, which were all significantly repressed by light exposure, were associated with functions including visual perception, phototransduction, cellular response to light stimulus, transcriptional regulation, and the regulation of DNA damage response. In addition, KEGG enrichment analyses revealed significant enrichment in pathways associated with phototransduction, MAPK signaling, DNA damage repair, and many key metabolic pathways across different clusters **(S5 Fig)**. To further understand the organization of these light regulated transcripts, we constructed co-expression networks for each cluster and identified hub genes using maximal clique centrality (MCC) values. The top 10 hub genes for each cluster, as well as the top hubs across all networks were identified (marked in red) **(Fig 2D)**. Intriguingly, the hub genes in cluster 1, which were rapidly up-regulated in response to light exposure, were predominantly clock genes, for example *cry1a*, *cry2*, *per2*, *per3* and *tefa*.

### 3.3 Characterization of light-responsive miRNAs in zebrafish larvae

**Fig 3:**
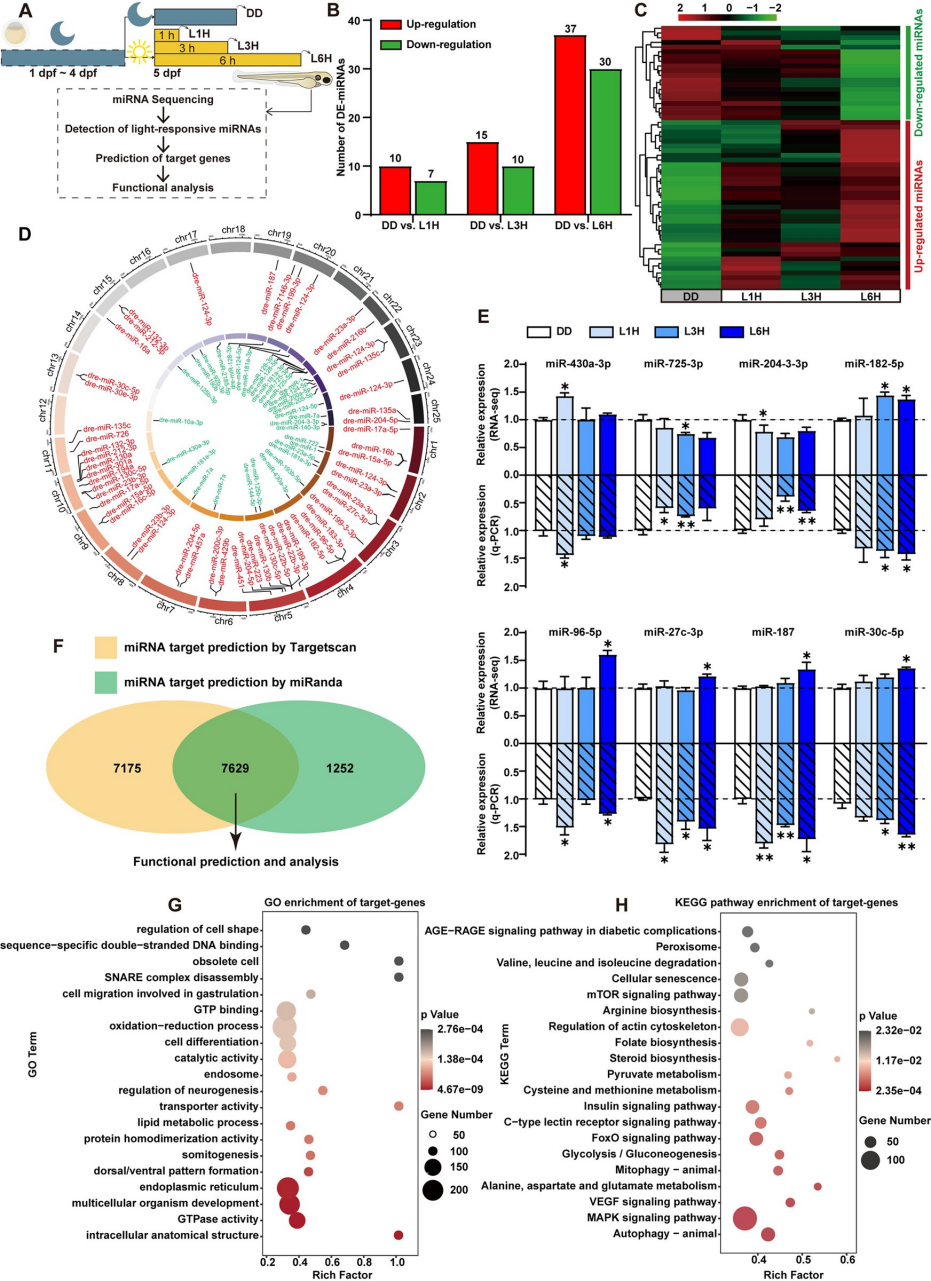
The light-responsive miRNAs in zebrafish larvae. **(A)** Flowchart illustrating the identification and expression profiling of light-responsive miRNAs in zebrafish larvae. **(B)** The number of light-responsive miRNAs identified in the comparisons between DD vs L1H, DD vs L3H, and DD vs L6H. **(C)** Heatmap displaying the expression levels of light-responsive miRNAs in zebrafish larvae, with colors ranging from red to green representing high to low expression levels, respectively. **(D)** Circular ideogram indicating the chromosomal locations of light-responsive miRNAs in zebrafish. **(E)** miRNA-sequencing and qRT-PCR verification of light-responsive miRNA expression. Values are presented as mean ± SEM in histograms. One-way ANOVA or Kruskal-Wallis test followed by multiple comparisons test results are reported in **S4 Table**. Significant differences are indicated by asterisks (***p < 0.001, **p < 0.01, *p < 0.05). **(F)** Venn diagram showing the overlap of miRNA-target genes predicted by TargetScan and miRanda. **(G-H)** GO and KEGG pathway analysis of the target genes of the light-responsive miRNAs. Bubble color and size correspond to the p-values and gene numbers enriched, respectively. The rich factor indicates the ratio of the number of miRNA-target genes mapped to a certain pathway to the total number of genes mapped to this pathway.

We next addressed the role played by miRNAs in shaping this light-induced mRNA expression profile. Specifically, we used miRNA-sequencing to search for light-responsive miRNAs using the same RNA samples prepared from the 5 dpf larvae raised in constant darkness and subsequently exposed to light for varying durations (0, 1, 3 and 6 h) **(Fig 3A)**. Most of the reads were 18–26 nt in length, with 22 nt miRNAs being the most abundant across all libraries **(S6 Fig)**. Among these zebrafish miRNAs, light-responsive miRNAs were screened via pairwise comparison with the DD control group, and a total of 17 miRNAs (10 up- and 7 down-regulated), 25 miRNAs (15 up- and 10 down-regulated) and 67 miRNAs (37 up- and 30 down-regulated) were identified in the L1H, L3H, L6H groups, respectively **(Fig 3B and S8 Table)**. Furthermore, according to their expression profiles across the different timepoints, we eliminated miRNAs which showed irregular patterns of expression and selected a final set of 66 light-responsive miRNAs, comprising 20 down-regulated and 46 up-regulated miRNAs **(Fig 3C)**. Genome-wide distribution analysis revealed that these light-responsive miRNAs were spread across 20 out of the 25 zebrafish chromosomes, with no distinct positional preferences **(Fig 3D)**. The light-responsive expression patterns of these miRNAs, as identified by miRNA-sequencing, were further validated by qRT-PCR **(Fig 3E)**. Importantly, gene expression analysis showed that dre-miR-430 family members which contain highly conserved seed regions, exhibited very similar expression patterns upon light exposure **(S7 Fig)**.

In order to understand the potential functions of these light-responsive miRNAs in zebrafish, bioinformatic analyses of their target binding sites were conducted via the TargetScan and miRanda databases, and 7629 predicted target genes were selected for functional annotation **(Fig 3F)**. Specifically, GO analysis of these miRNA target genes revealed significant enrichment in terms related to intracellular anatomical structure, GTPase activity, multicellular organism development, endoplasmic reticulum and oxidation-reduction processes **(Fig 3G)**. KEGG pathway annotation suggested that light-responsive miRNAs may regulate genes involved in MAPK signaling pathway, autophagy, VEGF signaling pathway, as well as the metabolism of alanine, aspartate and glutamate, some of which are known to be associated with light signal transduction and circadian rhythms in zebrafish **(Fig 3H)**.

### 3.4 Light-regulated mRNA-miRNA interaction networks

**Fig 4:**
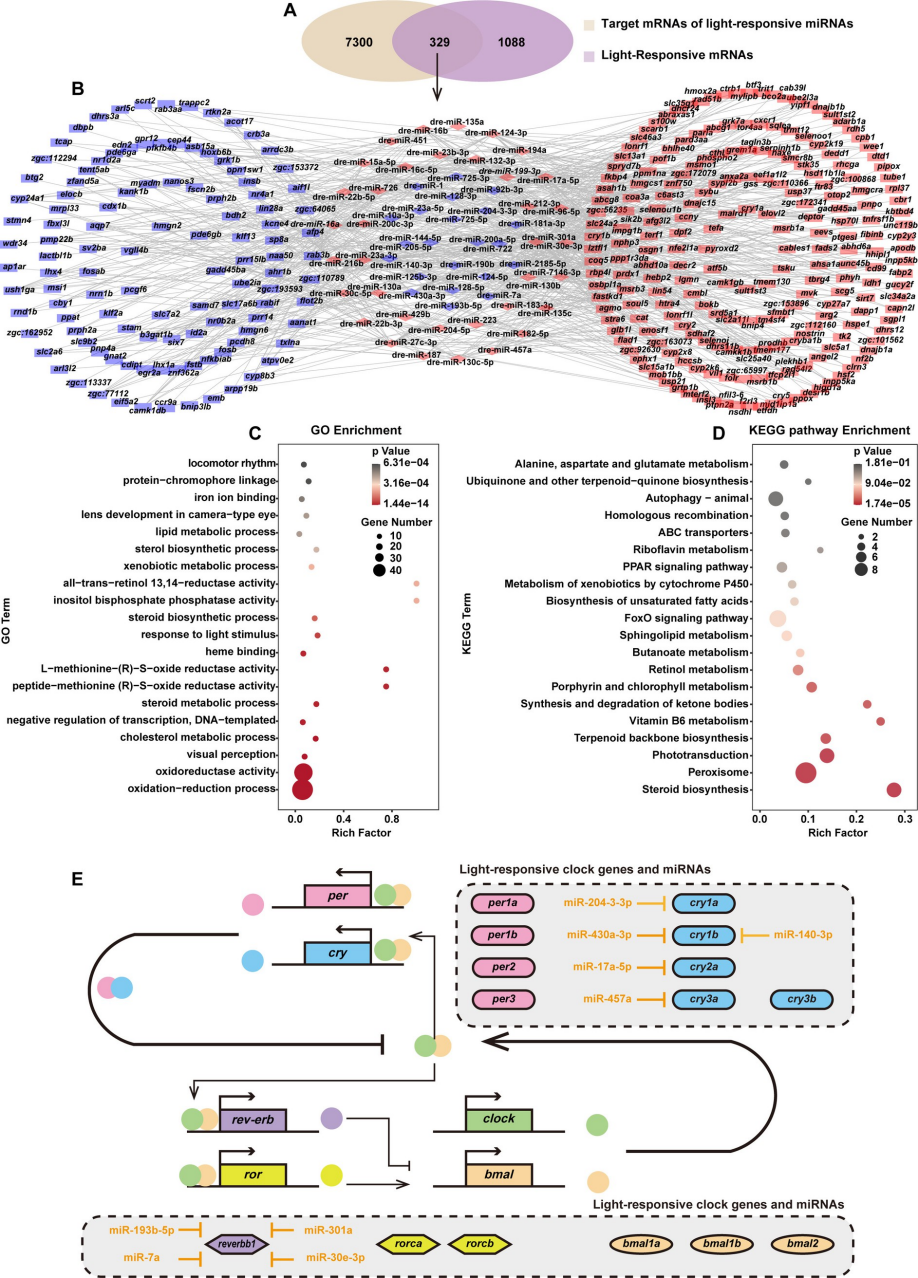
Construction of the light-responsive mRNA-miRNA interaction network. **(A)** Venn diagram showing the overlap between light-responsive genes and the targets of light-responsive miRNAs. **(B)** Interaction network of light-responsive mRNAs and miRNAs. Red nodes represent up-regulated RNAs and blue nodes represent down-regulated RNAs. **(C-D)** GO analysis and KEGG pathway enrichment of miRNA-target genes within the mRNA-miRNA interaction network. Bubble color and size correspond to the p-values and gene numbers enriched. The rich factor indicates the ratio of the number of miRNA-target genes mapped to a certain pathway to the total number of genes mapped to this pathway. **(E)** Proposed schematic diagram of the transcription-translation feedback loop in light-exposed zebrafish larvae, indicating the identified light-regulated clock genes and miRNAs within in the dotted box.

It is well established that miRNAs can regulate gene expression post-transcriptionally by binding to the 3′UTRs of their target mRNAs. Therefore, do light-regulated miRNAs play a role in shaping the dynamics of light-regulated mRNA expression? To explore the potential interaction between light-responsive miRNAs and mRNAs in zebrafish larvae, a conjoint analysis of their sequences was performed **(S8 Fig)**. By intersecting the predicted targets of light-responsive miRNAs with the identified light-responsive mRNAs, we constructed an interaction network that includes 329 light-responsive genes and 58 light-responsive miRNAs **(Fig 4A and 4B, S9 Table)**. Subsequently, these 329 genes within the regulatory network were searched against Gene Ontology database for function enrichment and the results included oxidation-reduction process, oxidoreductase activity, visual perception, transcriptional regulation as well as response to light stimulus and locomotor rhythm **(Fig 4C)**. KEGG pathway enrichment analysis indicated that this network may be linked to pathways involved in steroid biosynthesis, peroxisome function and phototransduction **(Fig 4D)**. Interestingly, within this light-responsive interaction network, several clock genes including *per*, *cry*, and *rev-erb*, which are integral components of the transcriptional-translational feedback loop (TTFL) of the circadian clock, were predicted to be modulated by multiple light-responsive miRNAs **(Fig 4E)**. This finding underscores the complex regulatory interplay between light-responsive miRNAs and the core circadian clock machinery.

### 3.5 Light-responsive miR-204 and miR-430a target 3′UTR of cryptochrome genes

**Fig 5:**
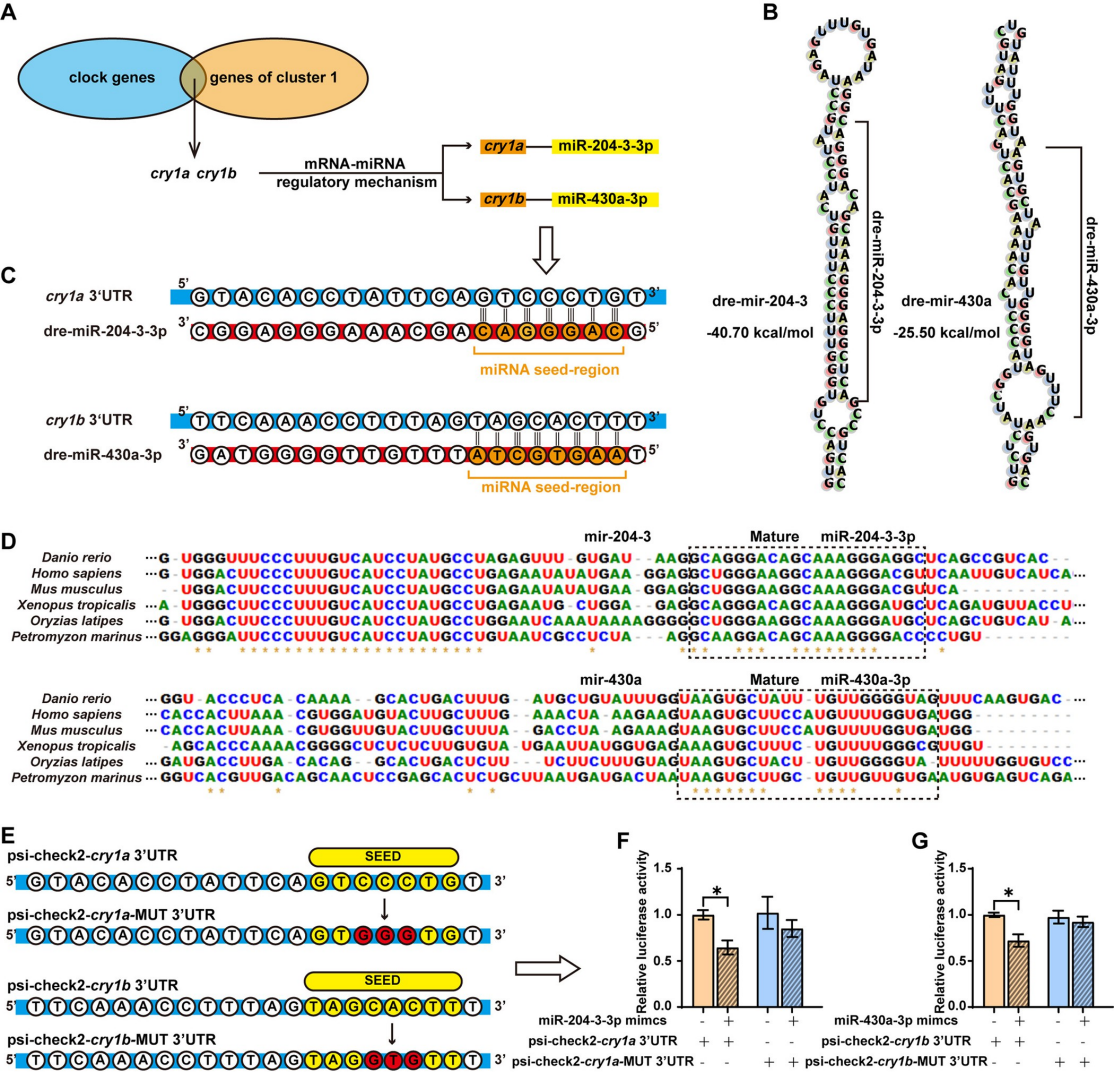
Light-responsive miR-204-3-3p and miR-430a-3p target cryptochrome genes. **(A)** Venn diagram showing that clock genes *cry1a* and *cry1b*, along with miR-204-3-3p and miR-430a-3p, were identified from the mRNA-miRNA interaction network and the K-means clustering analysis. **(B)** Predicted secondary structures of dre-miR-204 and dre-miR-430a precursors. **(C)** Sequences of miR-204-3-3p/miR-430a-3p and their potential binding sites in 3′UTRs of *cry1a*/*cry1b*. **(D)** Conservation of miR-204 and miR-430a precursors in various species. Alignments were performed using BioEdit 7.2.5, with asterisks indicating conserved sites among species. **(E)** Schematic representation of psicheck-2 constructs with inserts of the *cry1a* 3′UTR, *cry1a* mutation 3′UTR, *cry1b* 3′UTR and *cry1b* mutation 3′UTR. **(F-G)** Effects of miR-204-3-3p and miR-430a-3p on the luciferase expression of psicheck-2 plasmids containing the WT/Mut 3′UTR of *cry1a* and *cry1b*. The values are presented as mean ± SEM in histograms. One-way ANOVA followed by multiple comparisons test results are reported in **S4 Table**. Significant differences are indicated by asterisks (***p < 0.001, **p < 0.01, *p < 0.05).

To investigate the functional consequences of light-responsive miRNA-mRNA interactions, we focused on the network involving light-regulated clock genes. Specifically, two differentially light-responsive miRNAs, miR-204-3-3p which was down-regulated by light, and miR-430a-3p which was up-regulated by light **(Fig 3E)**, and their predicted direct target genes, *cry1a* and *cry1b*, were selected for further analysis **(Fig 5A)**. Initially, RNAfold was applied to predict the secondary structures of the precursor sequences for these miRNAs. Both miR-204-3-3p and miR-430a-3p precursors folded into typical hairpin structures **(Fig 5B)**. Subsequent sequence alignments using various tools revealed that the seed regions (5′ end of the mature miRNA) of miR-204-3-3p and miR-430a-3p were perfectly complementary to the 3′UTR of zebrafish *cry1a* and *cry1b*, respectively **(Fig 5C)**. Notably, a high degree of evolutionary conservation of these light-responsive miRNA sequences, as well as their target binding sites within the cryptochrome genes was observed across multiple vertebrate species including zebrafish (*Danio rerio*), humans (*Homo sapiens*), mice (*Mus musculus*), frog (*Xenopus tropicalis*), medaka (*Oryzias latipes*) and lamprey (*Petromyzon marinus*), highlighting the functional importance of these miRNAs **(Figs 5D and S9)**. To experimentally validate the interaction between these miRNAs and their target genes, we cloned the 3′UTR sequences of zebrafish *cry1a* and *cry1b*, either in their wild-type forms or with mutations designed to disrupt the miRNA binding sites, into the psi-check2 vector, downstream of the firefly luciferase reporter gene **(Fig 5E)**. Reporter plasmids and miRNA mimics were then co-transfected into HEK-293T cells. Our results showed that the miR-204-3-3p mimic significantly reduced the luciferase activity when paired with the wild-type *cry1a*-3′UTR, compared to a control mimic NC. However, no significant reduction in luciferase activity was observed when the miR-204-3-3p binding site in the reporter construct was mutated **(Fig 5F)**. Similar results were obtained when testing the interaction between miR-430a-3p and the *cry1b* 3′UTR **(Fig 5G)**. Thus, our results indicate that light-responsive miR-204-3-3p and miR-430a-3p directly target the clock genes *cry1a* and *cry1b*, respectively, through interactions with their 3′UTR binding sites.

### 3.6 Light-responsive miRNAs regulate circadian clock gene expression in zebrafish larvae

**Fig 6:**
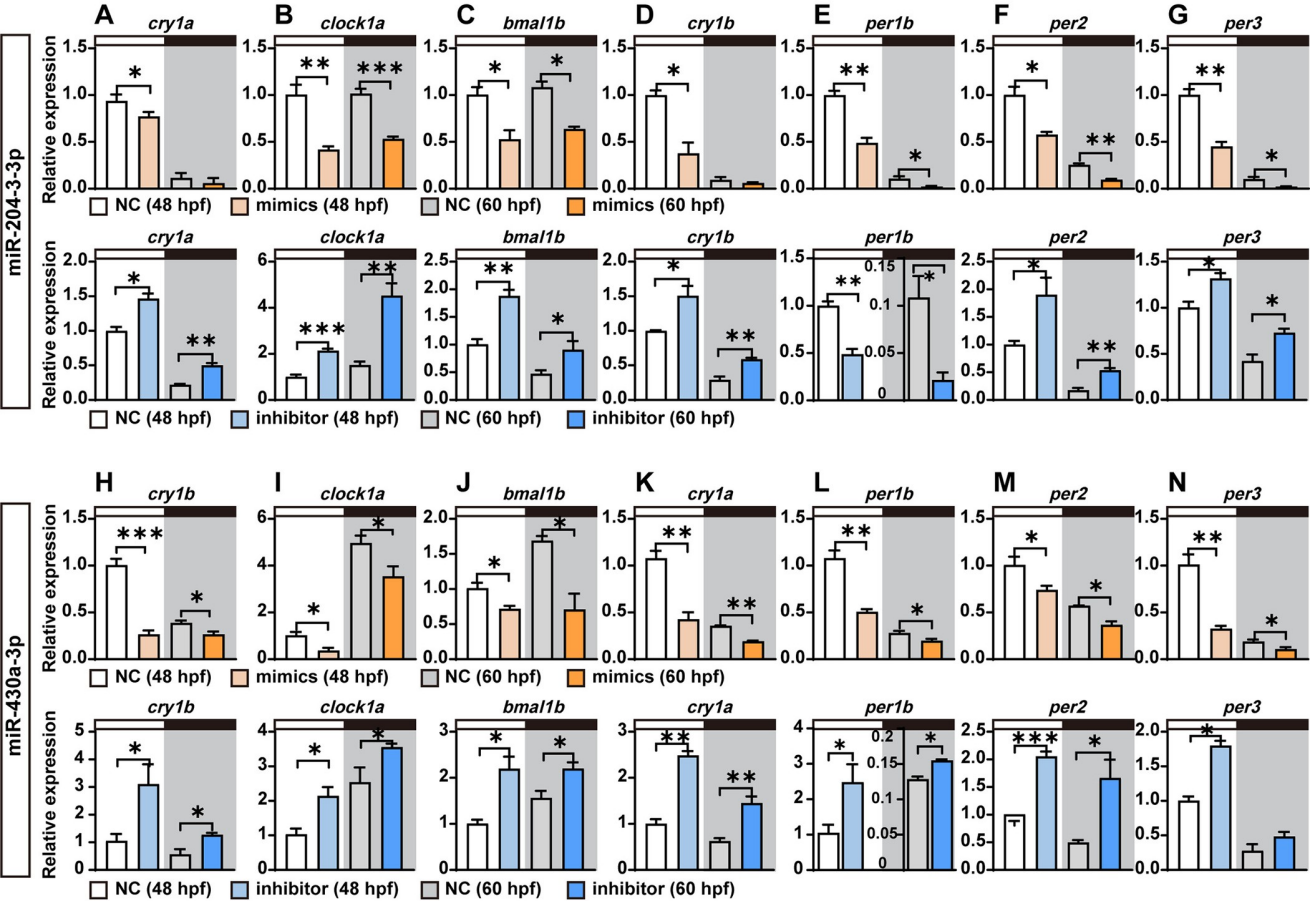
The effects of miR-204-3-3p and miR-430a-3p on the circadian clock machinery. **(A-G)** Effects of miR-204-3-3p mimics and inhibitors on the expression of key circadian clock components including *cryptochrome*, *period*, *bmal* and *clock* genes at different timepoints (48 hpf, light period; and 60 hpf, dark period). **(H-N)** Effects of miR-430a-3p mimics and inhibitors on the expression of key circadian clock components including *cryptochrome*, *period*, *bmal* and *clock* genes at different timepoints. Values are presented as mean ± SEM in histograms. One-way ANOVA or Kruskal-Wallis test followed by multiple comparisons test results are reported in **S4 Table**. Significant differences are indicated by asterisks (***p < 0.001, **p < 0.01, *p < 0.05).

The regulatory effects of these light-response miRNAs on *cryptochrome* mRNA expression suggest potential influences on the circadian clock machinery. We thus examined whether interference of miR-204-3-3p and miR-430a-3p has any effect on the expression of other core clock genes. Zebrafish embryos were microinjected at single- or two-cell stage with miRNA mimics, inhibitors or equivalent concentrations of a negative control (NC). The relative expression levels of clock genes at different time points (48 hpf and 60 hpf) were assessed using qRT-PCR. Interestingly, overexpression of miR-204-3-3p and miR-430a-3p significantly repressed not only their direct targets, *cry1a* and *cry1b*, respectively, but also markedly reduced the expression of the other core clock genes including *clock1a*, *bmal1b*, *per1b*, *per2* and *per3* at different time points **(Fig 6A–6N, upper panels)**. Conversely, inhibition of endogenous miR-204-3-3p and miR-430a/b/c-3p resulted in a significant upregulation of their target genes, as well as other clock components in 48 hpf (light period) and 60 hpf (dark period) embryos **(Fig 6A–6N, lower panels)**. The effects of miR-204-3-3p and miR-430a-3p mimics and inhibitors on clock gene expression appear to be very much gene-specific. In an extended panel of genes including DNA repair genes (*cry5* and *cry-DASH*), transcription factor regulators of the D-box enhancer (*dbpa*, *dbpb*, *tefa*, *nfil3-3* and *nfil3-6*), a clock regulated gene (*aanat1*) as well as other clock-regulatory genes (*nr1d2a* and *rorca*), we revealed differential effects of the mimic and inhibitor treatments **(S10 and S11 Figs)**. Thus, by comparing the results of overexpression and interference of these miRNAs, we speculate that these light-responsive miRNAs may play specific roles in circadian clock function in zebrafish. The differential regulation of clock genes by these miRNAs highlights the complexity of post-transcriptional control in circadian clock biology.

### 3.7 Light-responsive miRNAs modulate the rhythmic activity of zebrafish larvae

**Fig 7:**
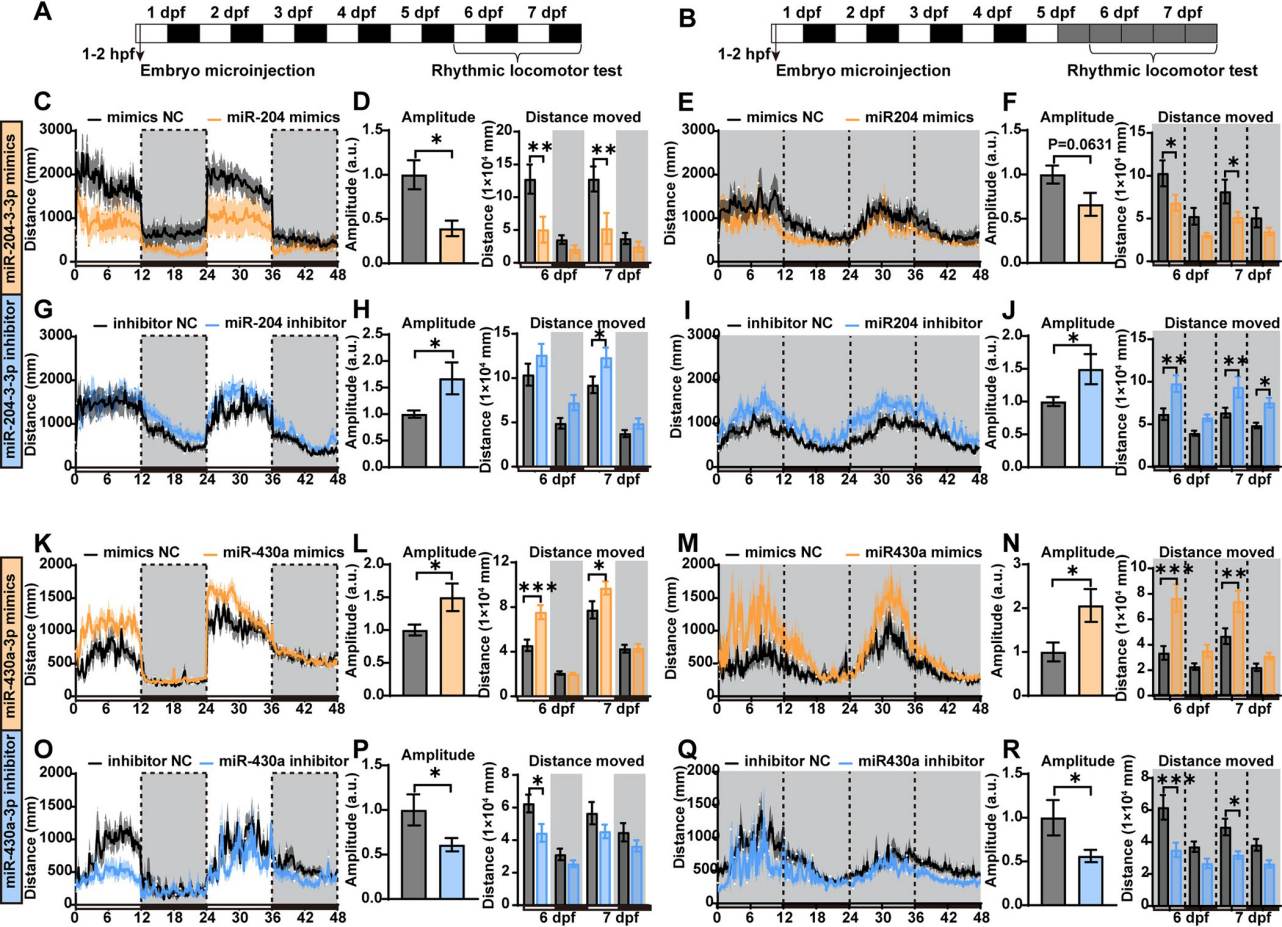
Light-responsive miRNAs modulate the rhythmic activity of zebrafish larvae. **(A-B)** Experimental designs for testing the rhythmic behavior of zebrafish larvae under light-dark (LD) cycle and constant darkness (DD, free-running period). Embryos were microinjected with miRNA mimics/inhibitors or NC at 2 hpf, and then were raised under LD for 5 days. The rhythmic locomotor activity of zebrafish larvae was monitored under LD (A) or DD (B) for two consecutive days. **(C-J)** Rhythmic locomotor activity of zebrafish larvae with miR-204-3-3p overexpressed or suppressed under LD or DD conditions. **(K-R)** Rhythmic locomotor activity of zebrafish larvae with miR-430a-3p overexpressed or miR-430a/b/c-3p suppressed under LD or DD conditions. Values are presented as mean ± SEM in line charts and histograms. Student’s t-test and one-way ANOVA followed by Dunnett’s multiple comparisons test results are reported in **S4 Table**. Significant differences are indicated by asterisks (***p < 0.001, **p < 0.01, *p < 0.05).

Rhythmic behavior is one of the most important outputs of the circadian clock. Given the effects of light-responsive miRNAs on core clock gene expression, we investigated whether modulation of these two selected miRNAs could influence the rhythmic locomotor activity of zebrafish larvae. Following previously established protocols [8,52], zebrafish embryos/larvae were injected with miRNA mimics or inhibitors, or equivalent concentrations of NC in order to confer miR-204-3-3p and miR-430a-3p overexpression or repression. Subsequently, their circadian clocks were entrained by five consecutive 24h day/night cycles, after which their locomotor activity was assessed between 6 and 7 dpf under either light/dark cycles or constant darkness (DD, free-running conditions) **(Fig 7A and 7B)**. Intriguingly, interference with miR-204-3-3p significantly affected the rhythmic locomotor activity of zebrafish larvae under both conditions. In comparison with the NC group, mimic-treated larvae displayed a reduced amplitude of rhythmic locomotor activity, along with decreased swimming distances, velocity and cumulative mobility **(Figs 7C–7F, S12A and S12B)**. In contrast, inhibitor-treated larvae exhibited increased amplitudes of rhythmic locomotor activity and generally higher levels of activity **(Figs 7G–7J, S12C and S12D)**. Interestingly, miR-204-3-3p and miR-430a-3p, which responded differently to light exposure, also appeared to have distinct effects on rhythmic behavior of zebrafish larvae. Specifically, miR-430a-3p mimics significantly enhanced the amplitude, swimming distance and velocity of larval rhythmic locomotor activity under both LD and DD conditions compared to the NC group **(Figs 7K–7N, S12E and S12F)**; Conversely, larvae treated with miR-430a-3p inhibitors showed reduced amplitude of rhythmic activity and lower activity levels during behavioral tests **(Figs 7O–7R, S12G and S12H)**. Thus, our behavioral data align with the observed effects on gene expression and point to light-responsive miRNAs playing a key role in shaping the rhythmic activity patterns of zebrafish larvae.

## 4. Discussion

Light is a critical regulator of circadian clocks across diverse organisms. While previous studies have extensively investigated the contribution of light-induced mRNA expression to circadian clock regulation, the involvement of light-responsive noncoding RNAs, particularly miRNAs, in this process remain unclear. The zebrafish constitutes a fascinating vertebrate model for studying the regulation of the circadian clockwork by light. Unlike the situation in mammals, the clocks in zebrafish peripheral tissues and cells are entrainable by direct light exposure thus providing unique insight into the function and evolution of light-dependent regulatory pathways. Therefore, with the goal of further exploring the intricate mechanisms underlying photic-entrainment of the circadian clock, we have characterized the regulatory network of light-responsive clock genes and noncoding miRNAs in whole zebrafish larvae. We have identified miRNAs which target two cryptochrome gene 3′UTR regions and thereby regulate mRNA levels for both clock gene transcripts. Interestingly, manipulating the activity of these miRNA by the use of artificial mimics and inhibitors has a broader, but specific effect on the expression levels of several other clock genes as well as the profile of clock regulated locomotor activity in zebrafish larvae. These results extend our understanding of the multilevel effects of light on gene regulatory networks associated with the circadian clock.

### 4.1 Transcriptional characterization of light-responsive genes in zebrafish larvae

Our previous studies have demonstrated the crucial role of the D-box cis-acting enhancer element in directing light-driven gene transcription in zebrafish [18,21,53]. Light exposure increases intracellular ROS levels, which in turn activate the JNK and p38 MAP kinase signaling pathways, inducing gene transcription through the activation of the D-box-binding PAR/E4BP4 class of bZip transcription factors [54]. To date, many clock genes and DNA repair genes including *per2*, *cry1a*, *cry5* and *ddb2* have been identified as light-responsive and containing multiple D-boxes in their promoter regions [18,53,55]. To obtain a broader picture of light-responsive gene regulatory networks in zebrafish, we characterized the transcriptome of zebrafish larvae exposed to light via RNA-sequencing and identified 1365 light-responsive genes. The DD control group was sampled at a single timepoint, midway through the 6h period of light exposure. Therefore, in addition to the effects of light exposure, some limited differences in the developmental stages of the larvae being compared may also contribute to some of the observed changes in gene expression. GO enrichment analyses revealed significant enrichment in the oxidation-reduction process, consistent with the altered redox balance upon light exposure, and the results of previous studies demonstrating that oxidative stress and ROS serve as key factors in many light-regulated biological processes [56,57] including the entrainment of circadian clocks by light [58,59]. Among the top 20 enriched KEGG pathways, biosynthetic and metabolic pathways including steroid biosynthesis, metabolism of arachidonic acid, retinol, glutathione and enzymes were significantly enriched, which is consistent with previous findings indicating the strong involvement of ambient light in metabolic regulation [60–65]. Importantly, the PPAR signaling pathway was also significantly enriched in our functional enrichment analysis, which has been known to regulate circadian rhythms by interacting with core clock components [66–68]. For example, PPARγ directly interacts with core clock genes including *per2* and *nrqd1* [69,70], and abnormal expression of *PPARγ* has been shown to disrupt diurnal rhythms in mice [71].

### 4.2 Light-responsive miRNAs in zebrafish larvae

Increasing evidence supports the significant role of post-transcriptional mechanisms in circadian clock regulation [72–77]. Generally, miRNAs regulate expression of their target genes through transcript cleavage or translation repression, leading to decreased levels of their mRNA targets [24,78]. Here, we have revealed that light-inducible miRNAs in zebrafish are involved in circadian clock regulation. GO and KEGG analyses showed significant enrichment of genes involved in MAPK signaling pathway and oxidation-reduction processes among the targets of these light-responsive miRNAs. According to previous studies, redox changes stimulate intracellular MAPK signaling, triggering the transduction of photic signals [79,80], while the roles of MAPK signaling pathway in circadian clock regulation have been established in many organisms [79,81,82]. Thus, our results imply that light-responsive miRNAs may participate in the regulation of zebrafish circadian clocks by mediating oxidation-reduction homeostasis and the MAPK signaling pathway, or alternatively, by directly interacting with key circadian clock genes.

We have identified two light-responsive miRNAs which target the 3′UTR regions of *cry1a* and *cry1b* transcripts, key negative regulators of the core TTFL in the circadian clock. Our analyses of endogenous gene expression demonstrated that interference with these two miRNAs not only significantly repressed the expression of their target genes, but also indirectly affected many other components of the circadian clock which do not possess predicted binding sites for either of the light-regulated miRNAs. The specificity of these global effects on circadian clock gene expression is further corroborated by consistent changes in the amplitude of circadian rhythms of locomotor activity in zebrafish larvae treated with miRNA mimics or inhibitors. Furthermore, other non-core clock genes are differentially affected or unaffected by mimic or inhibitor treatment, suggesting a gene-specific regulatory mechanism. The exact mechanisms linking miR-204-3-3p and miR-430a-3p with the regulation of clock genes which lack their 3′UTR binding sites is currently unclear. However, it is tempting to speculate that these miRNAs participate in a larger regulatory network of miRNAs which may collectively target a broader set of clock gene regulatory targets.

The circadian clock governs the rhythmic behavior of organisms, and aberrant expression of clock genes can modulate their rhythmic activities [83]. Importantly, previous studies have demonstrated severe morphological defects in zebrafish embryos following complete genetic loss of the miR-430 family, whether via Dicer loss-of-function or miR-430 locus deletion. However, our results revealed that microinjection of miRNA inhibitors at our selected doses did not induce either observable malformation, or gross changes in locomotor activity and swimming posture, suggesting that the effective knockdown achieved by our antisense inhibitors as verified by qRT-PCR analysis **(S13 Fig)** was likely partial compared to a complete genetic loss-of-function. Interestingly, although the phases of their rhythmic activity remained unchanged, the amplitude and overall robustness were significantly altered in larvae treated with miRNA mimics or inhibitors under both LD and DD conditions. Our findings provide important clues as to the molecular mechanisms by which miR-204-3-3p and miR-430a-3p regulate circadian behavior in zebrafish. The dysregulation of *cry1a* and *cry1b* by these miRNAs mimics/inhibitors disrupts the transcription-translation feedback loop (TTFL) of the circadian clock, leading to quantitative and qualitative alterations in the expression patterns of a range of core clock genes. In addition, the Cry1a and Cry1b proteins play a role in repressing the activity of CLOCK-BMAL1 complexes, thereby regulating the amplitude of rhythmic expression of downstream clock-controlled genes as well as circadian rhythms in various physiological processes [84,85]. In turn, this disruption results in changes in circadian rhythm amplitudes, as revealed by the abnormal locomotor activity observed in zebrafish larvae. Importantly, although miR-204-3-3p and miR-430a-3p target a pair of homologous genes in zebrafish, we observed distinct behavioral effects upon interference of these two miRNAs. These differences may be attributed to the differential light-responsive expression patterns and dynamics of these miRNAs (miR-204-3-3p is down-regulated by light, whereas miR-430a-3p is up-regulated), their differential target specificity and regulation (additional targets that may account for the distinct phenotypic outcomes), mechanistic differences in miRNA action (the balance between mRNA degradation and translational repression), as well as the functional differences between their target genes. The divergence in the roles of *cry1a* and *cry1b* likely reflects the consequence of genome duplication events that occurred early during teleost evolution [86–88], resulting in a series of zebrafish cryptochrome genes (*cry1a*, *cry1b*, *cry2a*, *cry2b*, *cry3*, *cry4* and *cry5*) with varying evolutionary relationships and biological functions [89,90]. For example, *cry1a* is a light-responsive gene directly induced by light exposure, while *cry1b* is mainly clock regulated [15]. In addition, these miRNAs likely target multiple genes, suggesting that the observed modulation of rhythmic behavior in zebrafish larvae may result from interference with other biological functions and pathways. For example, miR-204 is widely expressed in the eye, bone, cardiovascular and central nervous systems and has been implicated in various cancers and ocular diseases [91–93]. In contrast, miR-430 has been reported to exhibit high-expression levels during zebrafish early development [94], playing roles in cell division, cardiac development and neurodevelopment [94–97]. Therefore, these light-regulated miRNAs represent potential points of crosstalk between the circadian clock, photic responses and other physiological systems.

Comparing our whole-larvae microRNA sequencing data and the microarray results from the zebrafish pineal gland [22], we have identified 7 overlapping light-regulated miRNAs: miR-96, miR-182, miR-183, miR-212, miR-125b, miR-140 and miR-27c. Notably, miR-96, miR-182, and miR-183 are members of the miR-183 cluster (miR-183C), suggesting that these three light-responsive microRNAs are produced from the same primary transcript [98,99]. This microRNA cluster is highly expressed in the nervous system and has been identified as a modulator of circadian rhythms, affecting the expression of core circadian clock genes [100,101]. Therefore, our findings in whole zebrafish larvae further validate previous results obtained from other tissues and organisms, suggesting conserved regulation and function of the miR-183 cluster across multiple species. Furthermore, miR-212 is highly expressed in the nervous system, where it plays roles in neuroprotection and neurodevelopment [102,103]; miR-27c has been reported to affect the innate immune system, regulate mitochondrial networks by targeting genes governing mitochondrial dynamics, and to be involved in neuronal survival and axon growth during repair process [104–106]; miR-125b is a key regulator of heat stress response and metabolic processes [107,108], while miR-140 is implicated in pulmonary arterial hypertension and cartilage integrity [109,110]. The conserved, direct and indirect light-responsiveness of these miRNAs serves as an additional validation of our current and previous results and underscores the importance of light-responsive miRNAs in the adaptation of zebrafish physiology and behavior to the day-night cycle.

## 5. Conclusion

**Fig 8:**
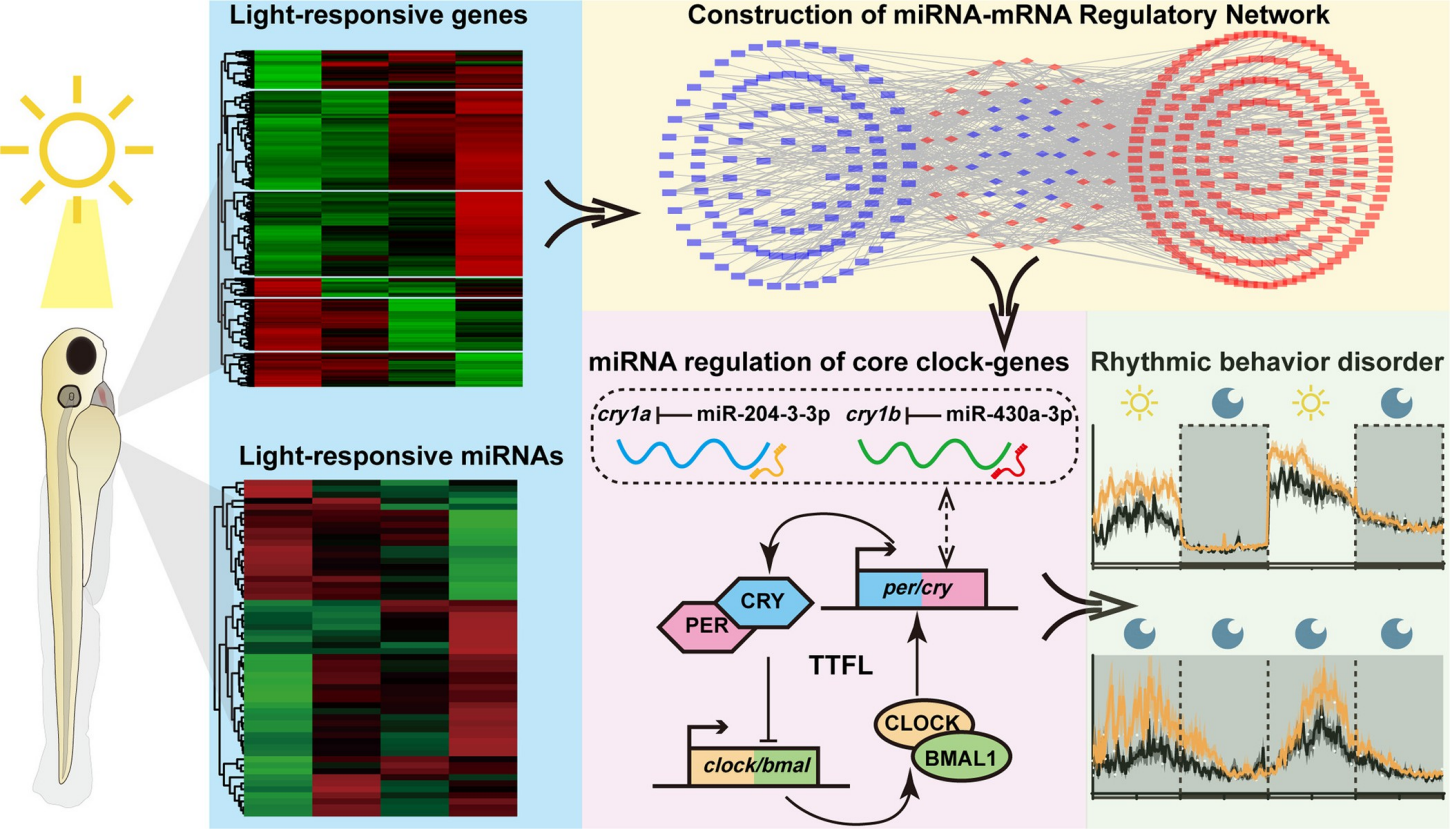
Graphic illustration: Identification and functional characterization of light-responsive miRNAs in zebrafish larvae.

In this study, we used RNA sequencing to characterize light-responsive gene regulatory networks in zebrafish larvae and have identified 1365 light-regulated mRNAs involved in processes including oxidation-reduction, visual perception, locomotor rhythm, PPAR signaling, peroxisome function and phototransduction. Additionally, we have identified 66 light-responsive miRNAs predicted to modulate many biological events including MAPK signaling and peroxisome function, demonstrating the dual role of light in regulating zebrafish circadian clocks at both the transcriptional and post-transcriptional levels. Based on this data we constructed a light-responsive mRNA-miRNA interaction network and selected two miRNAs for functional validation. Specifically, we demonstrated that miR-204-3-3p and miR-430a-3p, which target the 3′UTRs of two core clock components (*cry1a* and *cry1b*), regulate not only the core clock machinery but also rhythmic behavior of zebrafish larvae **(Fig 8)**. Thus, our expression profiling and functional characterization of light-responsive mRNAs and miRNAs has verified an intricate, multi-level modulation of the circadian clockwork by light, and provided new insights into how non-coding RNA regulation potentially enables crosstalk between the circadian clock, photic responses and other physiological systems in the zebrafish.

## Supporting information

S1 Table Primer sequences used for qRT-PCR analysis. (XLSX)

S2 Table Sequences of miRNA mimics and inhibitors. (XLSX)

S3 Table Primer sequences used for vector construction and site-directed mutagenesis. (XLSX)

S4 Table Detailed numerical data for graphs and summary statistics in this study. (XLSX)

S5 Table The quality and quantity of RNA samples used for transcriptome sequencing. (XLSX)

S6 Table Summary of RNA-seq read counts. (XLSX)

S7 Table Expression profiles of light-responsive mRNAs in zebrafish larvae. (XLSX)

S8 Table Expression profiles of light-responsive miRNAs in zebrafish larvae. (XLSX)

S9 Table Details information of light-responsive mRNA-miRNA interaction network. (XLSX)

S1 Fig Schematic workflow of mRNA-seq and miRNA-seq in this study. (PDF)

S2 Fig Analysis of total RNA integrity. (PDF)

S3 Fig Overview of the mRNA-seq data indicating FPKM density distribution. (PDF)

S4 Fig The verification of mRNA-seq data by qRT-PCR analysis. (PDF)

S5 Fig KEGG pathway enrichment analysis of each light-responsive gene cluster. (PDF)

S6 Fig Length distribution of sequences in zebrafish small RNA-seq libraries. (PDF)

S7 Fig Expression profile and multiple sequence alignment of miR-430 family members. (PDF)

S8 Fig Flow chart of the light-responsive miRNA-mRNA integrative analysis. (PDF)

S9 Fig miRNA target sites in *cry* genes of zebrafish and the other species. (PDF)

S10 Fig The effects of miR-204-3-3p on the other circadian clock and DNA repair genes. (PDF)

S11 Fig The effects of miR430a-3p on the other circadian clock and DNA repair genes. (PDF)

S12 Fig Light-responsive miRNAs modulate the rhythmic activity of zebrafish larvae. (PDF)

S13 Fig qRT-PCR analysis of miR-430 abundance in zebrafish larvae upon microRNA inhibitor microinjection. (PDF)
