# Supplementary figures and images for: Light-regulated microRNAs shape dynamic gene expression in the zebrafish circadian clock
Source: PLoS Genet. 2025 Jan 8;21(1):e1011545. doi: 10.1371/journal.pgen.1011545 (PMC11750094; doi:10.1371/journal.pgen.1011545)

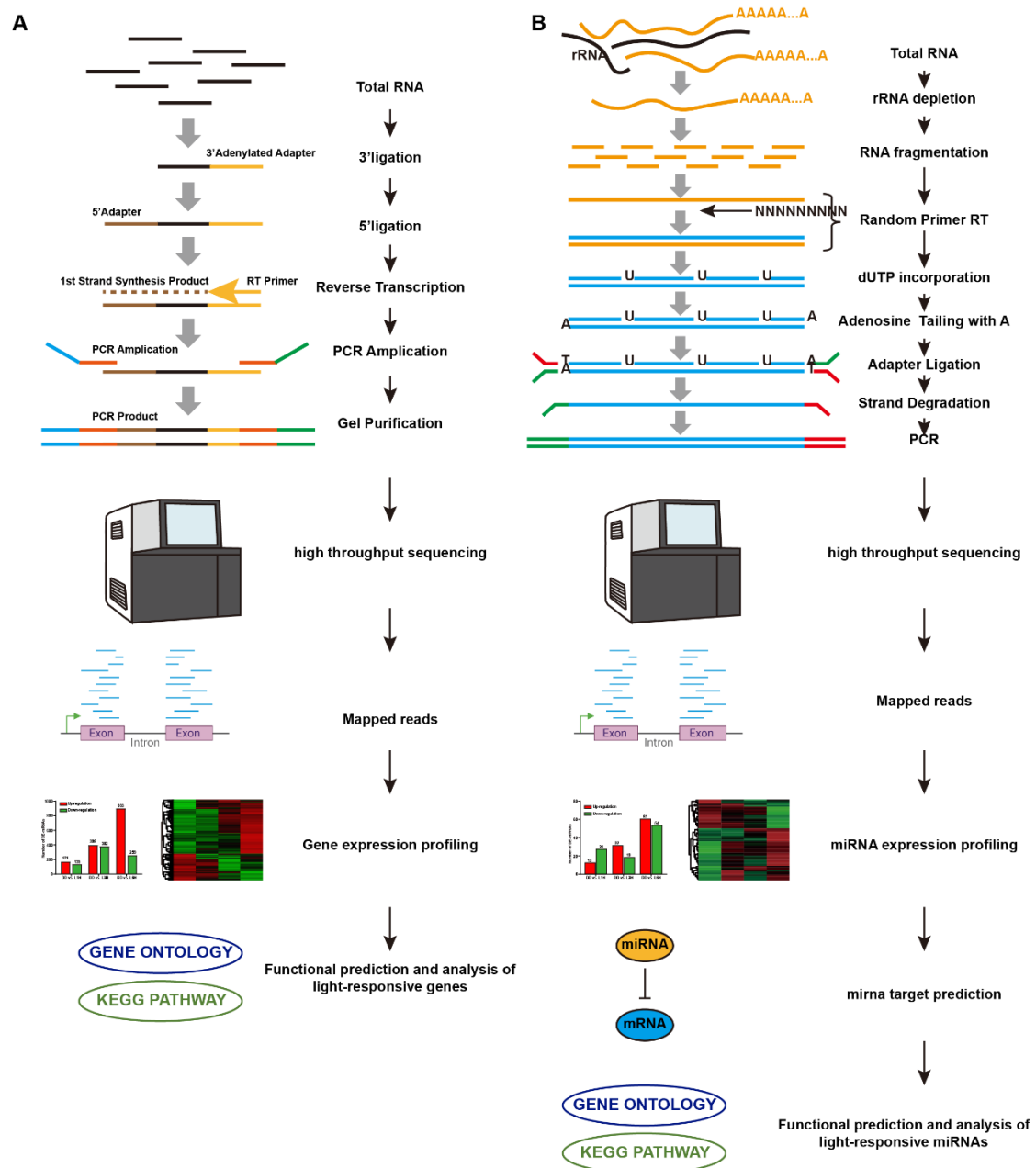

**S1 Fig. Schematic workflow of mRNA-seq and miRNA-seq in this study.**

Supplement: S1 Fig — (PDF) [file pgen.1011545.s010.pdf]

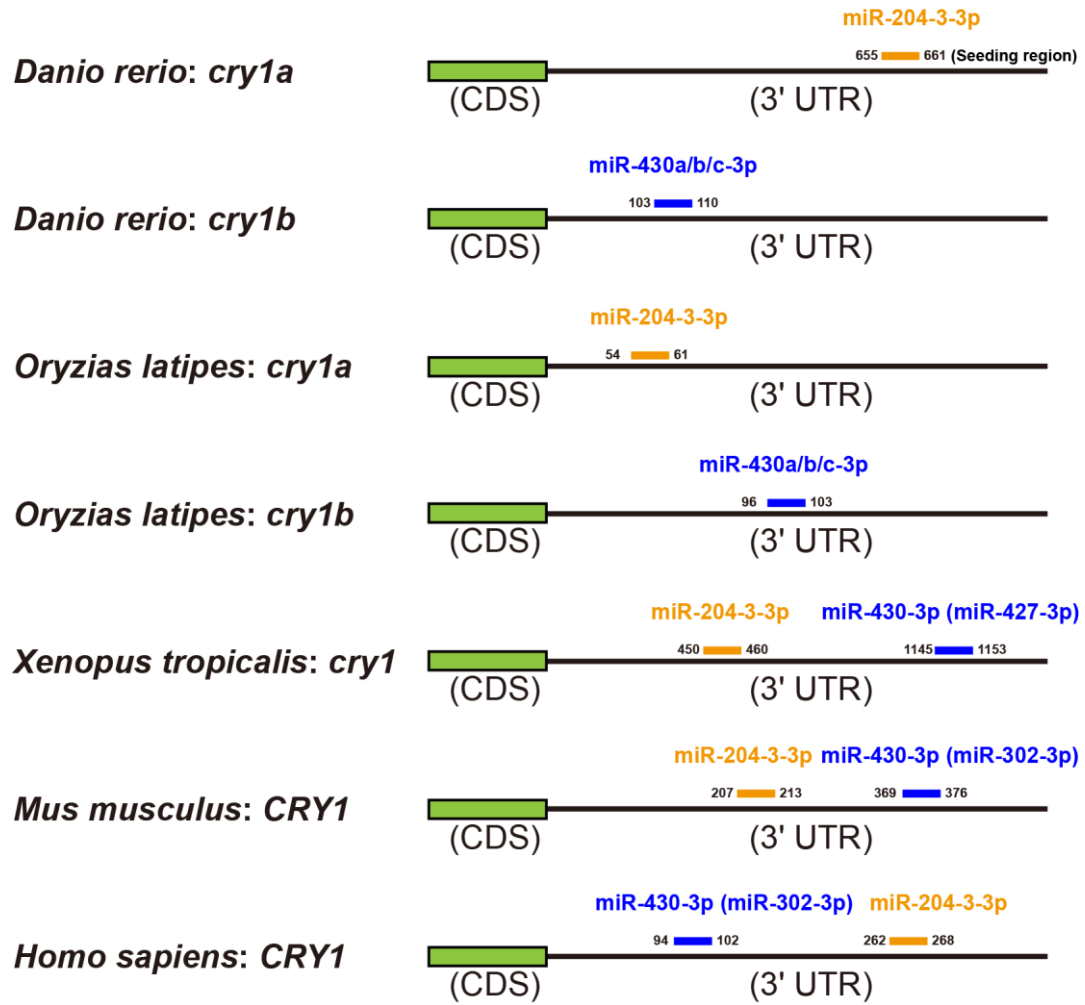

**S9 Fig. miRNA target sites in cry genes of zebrafish and the other species.**

Supplement: S9 Fig — (PDF) [file pgen.1011545.s018.pdf]
