# Supplementary material for: Light-regulated microRNAs shape dynamic gene expression in the zebrafish circadian clock
Source: PLoS Genet. 2025 Jan 8;21(1):e1011545. doi: 10.1371/journal.pgen.1011545 (PMC11750094; doi:10.1371/journal.pgen.1011545)
Supplement: S2 Fig — (PDF) [file pgen.1011545.s011.pdf]

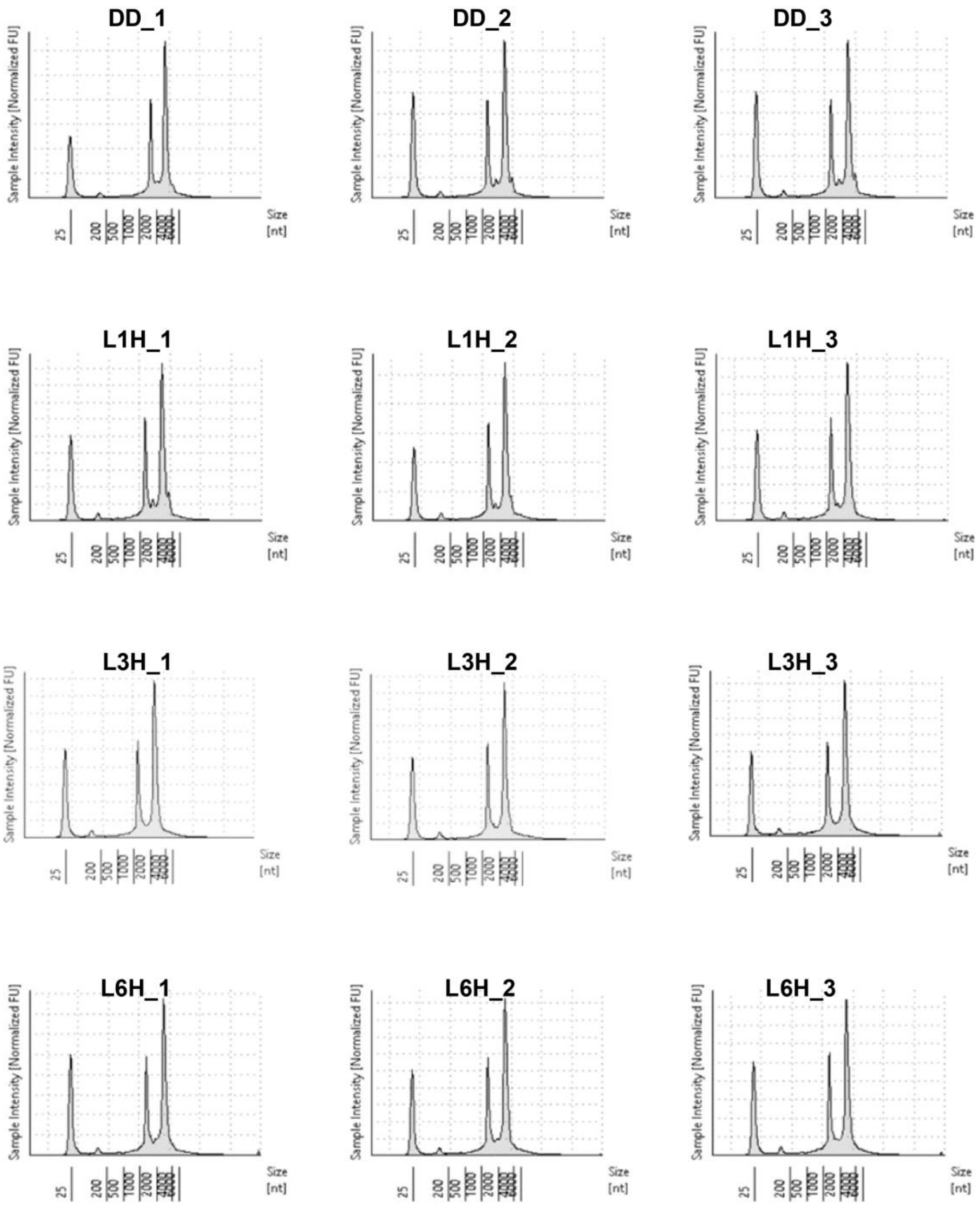

S2 Fig. Analysis of total RNA integrity. Agilent bioanalyzer chromatograms of total RNA in each sample.
