# Supplementary material for: Light-regulated microRNAs shape dynamic gene expression in the zebrafish circadian clock
Source: PLoS Genet. 2025 Jan 8;21(1):e1011545. doi: 10.1371/journal.pgen.1011545 (PMC11750094; doi:10.1371/journal.pgen.1011545)
Supplement: S3 Fig — (PDF) [file pgen.1011545.s012.pdf]

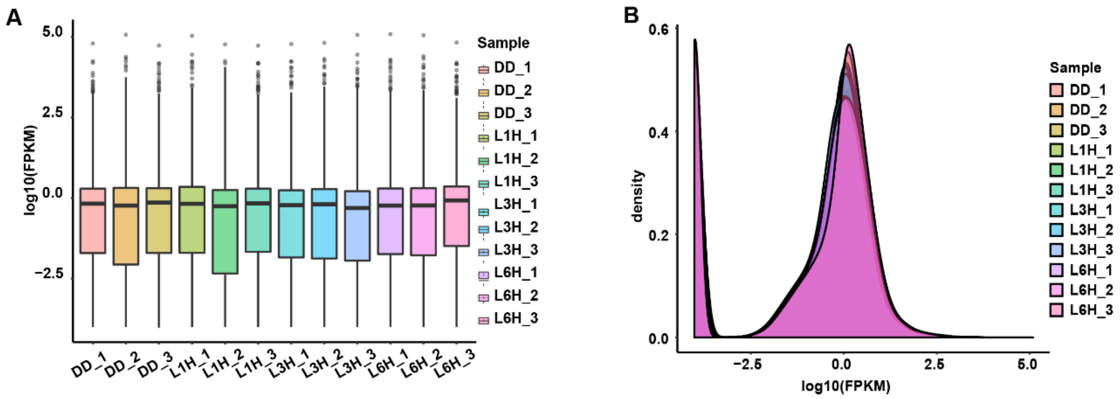

S3 Fig. Overview of the mRNA-seq data indicating FPKM density distribution. (A) Boxplot of the FPKM density distribution in each sample. **(B)** FPKM density distribution of the mRNA-sequencing data. The x-axis represents the log10 (FPKM) value of the gene, and the y-axis represents the distribution density of the genes with corresponding expression.
