# Supplementary material for: Light-regulated microRNAs shape dynamic gene expression in the zebrafish circadian clock
Source: PLoS Genet. 2025 Jan 8;21(1):e1011545. doi: 10.1371/journal.pgen.1011545 (PMC11750094; doi:10.1371/journal.pgen.1011545)
Supplement: S4 Fig — (PDF) [file pgen.1011545.s013.pdf]

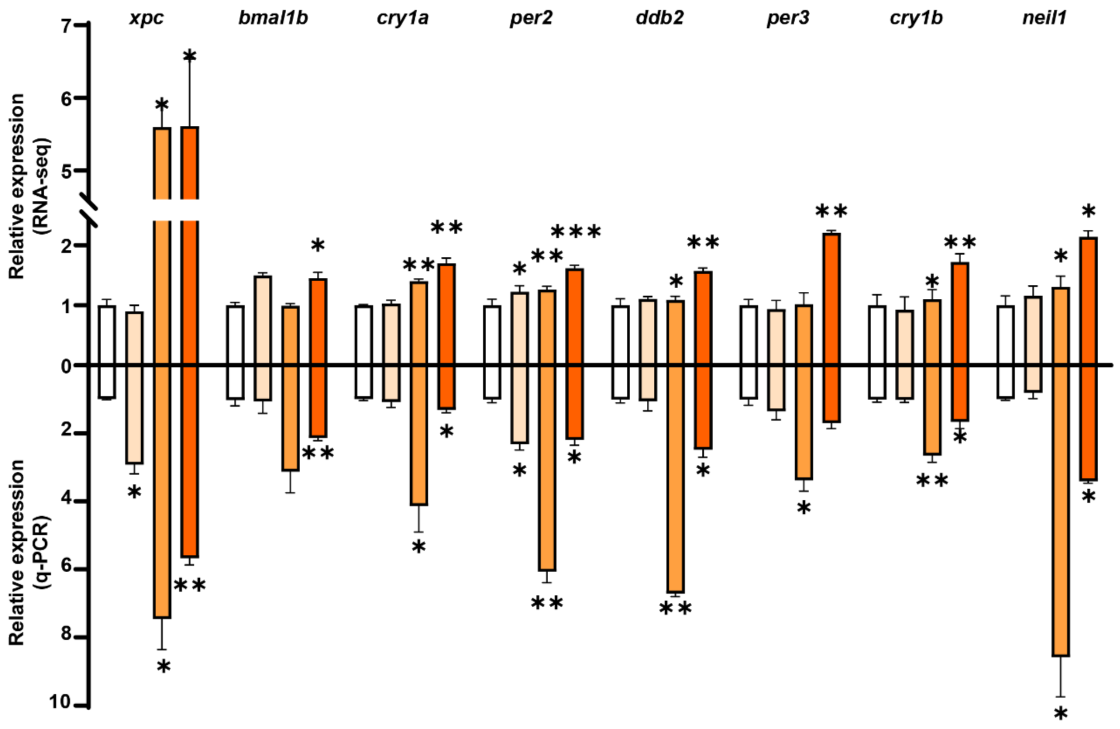

S4 Fig. The verification of mRNA-seq data by qRT-PCR analysis. RNA-Seq transcriptome analysis and qRT-PCR verification of the light-responsive circadian clock genes and DNA repair genes. The values are presented as mean ± SEM in histograms. One-way ANOVA or Kruskal-Wallis test followed by multiple comparisons test results are reported in **S4 Table**. Significant differences are indicated by asterisks (***p < 0.001, **p < 0.01, *p < 0.05).
