# Supplementary material for: Light-regulated microRNAs shape dynamic gene expression in the zebrafish circadian clock
Source: PLoS Genet. 2025 Jan 8;21(1):e1011545. doi: 10.1371/journal.pgen.1011545 (PMC11750094; doi:10.1371/journal.pgen.1011545)
Supplement: S5 Fig — (PDF) [file pgen.1011545.s014.pdf]

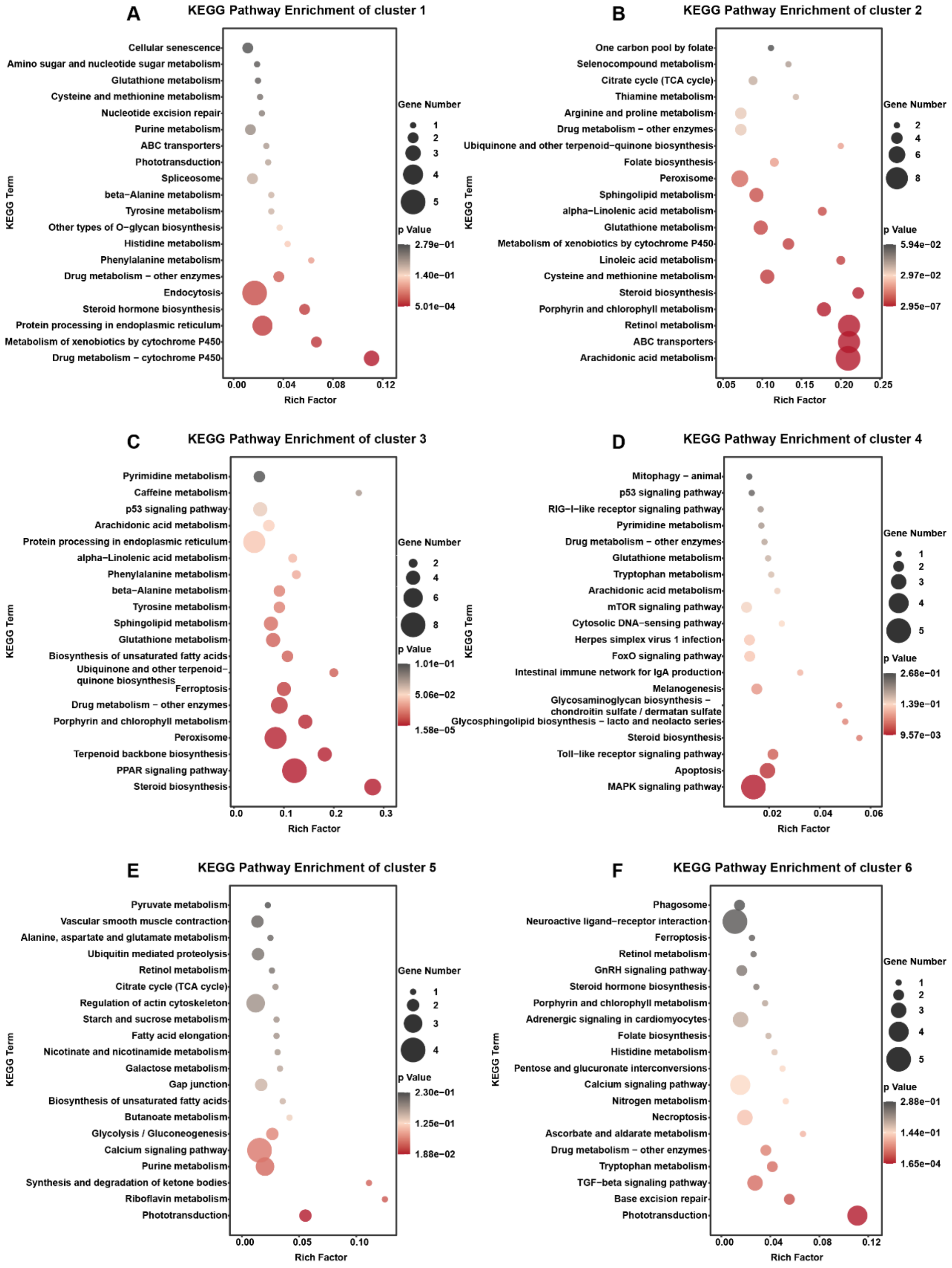

S5 Fig. KEGG pathway enrichment analysis of each light-responsive gene cluster. Bubble size indicates the number of genes enriched in each pathway, with color intensity corresponding to the p-value.
