# Supplementary material for: Light-regulated microRNAs shape dynamic gene expression in the zebrafish circadian clock
Source: PLoS Genet. 2025 Jan 8;21(1):e1011545. doi: 10.1371/journal.pgen.1011545 (PMC11750094; doi:10.1371/journal.pgen.1011545)
Supplement: S6 Fig — (PDF) [file pgen.1011545.s015.pdf]

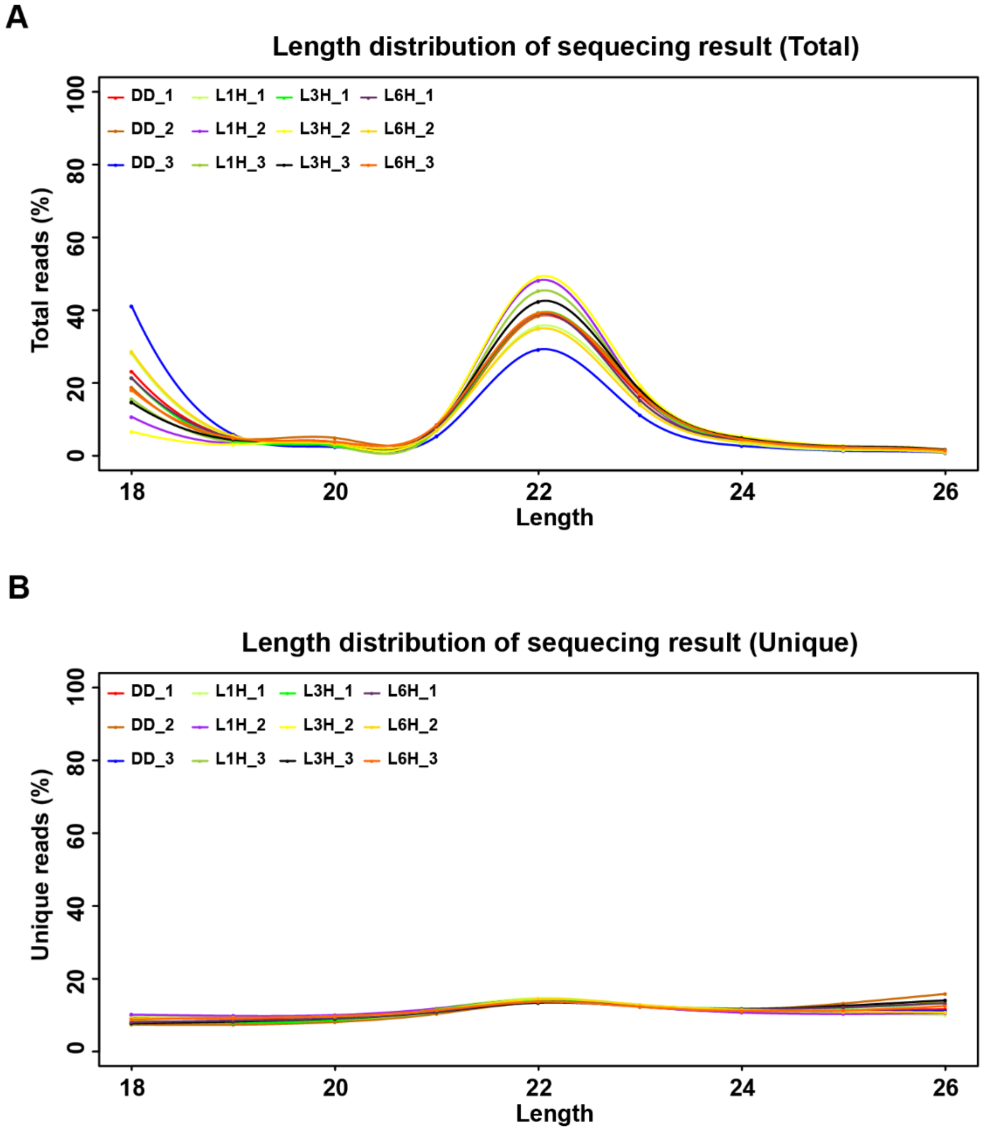

S6 Fig. Length distribution of sequences in zebrafish small RNA-seq libraries. (A) Length distribution of total reads. **(B)** Length distribution of unique reads.
