# Supplementary material for: Light-regulated microRNAs shape dynamic gene expression in the zebrafish circadian clock
Source: PLoS Genet. 2025 Jan 8;21(1):e1011545. doi: 10.1371/journal.pgen.1011545 (PMC11750094; doi:10.1371/journal.pgen.1011545)
Supplement: S7 Fig — (PDF) [file pgen.1011545.s016.pdf]

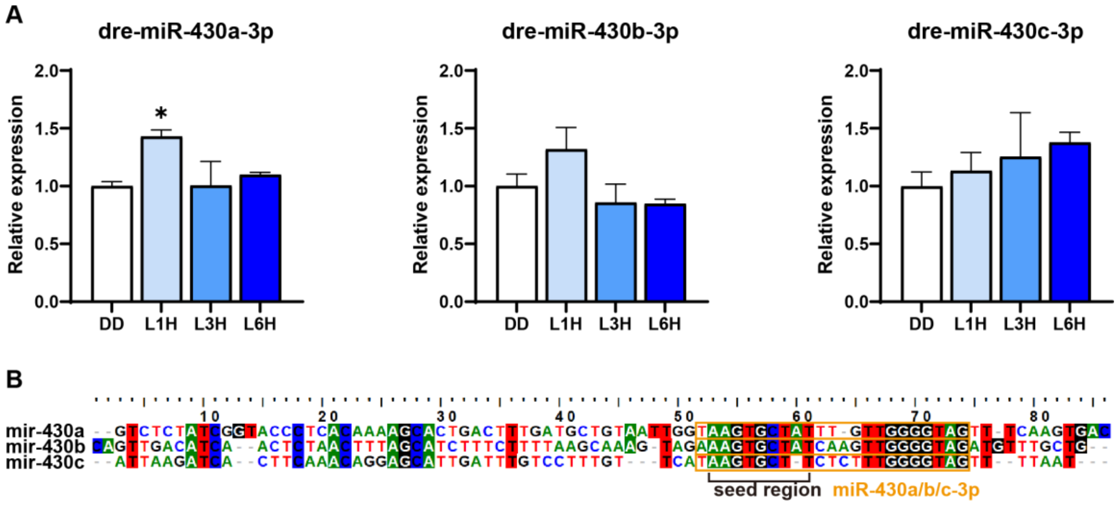

S7 Fig. Expression profile and multiple sequence alignment of miR-430 family members. (A) Light-responsive expression patterns of miR-430 family members. **(B)** Sequence alignment of miR-430 family members in zebrafish.
