# Supplementary material for: Light-regulated microRNAs shape dynamic gene expression in the zebrafish circadian clock
Source: PLoS Genet. 2025 Jan 8;21(1):e1011545. doi: 10.1371/journal.pgen.1011545 (PMC11750094; doi:10.1371/journal.pgen.1011545)
Supplement: S8 Fig — (PDF) [file pgen.1011545.s017.pdf]

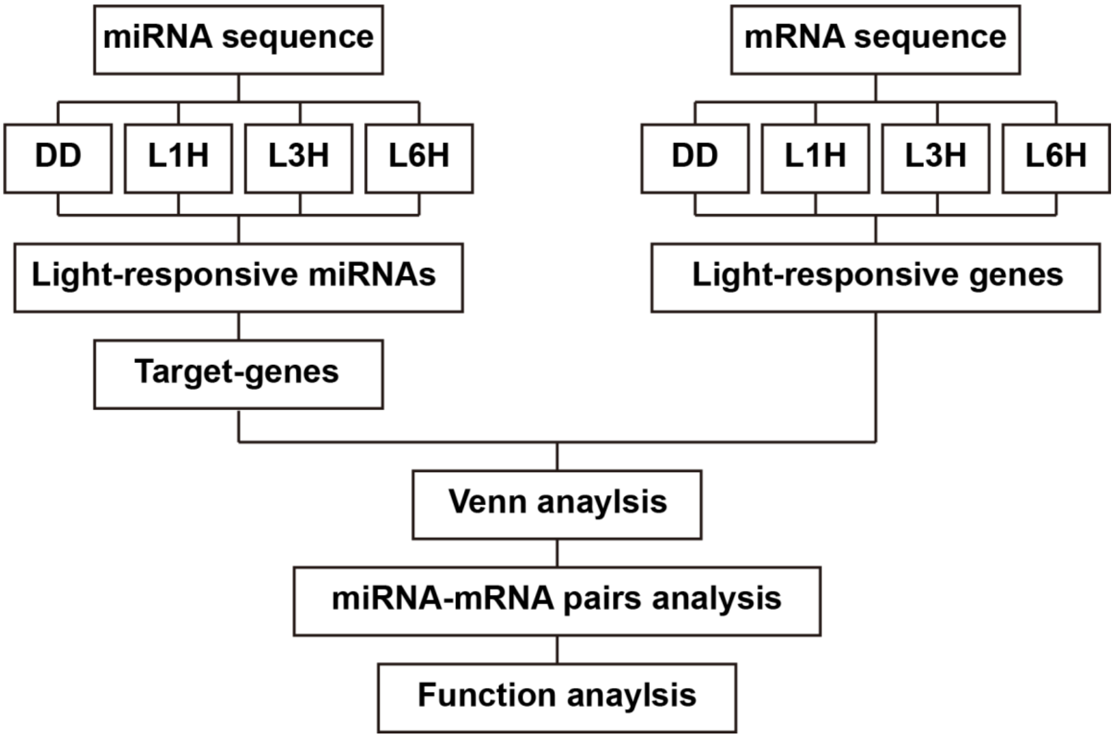

S8 Fig. Flow chart of the light-responsive miRNA-mRNA integrative analysis. Schematic flow diagram of the proposed approach for identification of the light-responsive miRNA-mRNA modules.
