# Supplementary material for: Light-regulated microRNAs shape dynamic gene expression in the zebrafish circadian clock
Source: PLoS Genet. 2025 Jan 8;21(1):e1011545. doi: 10.1371/journal.pgen.1011545 (PMC11750094; doi:10.1371/journal.pgen.1011545)
Supplement: S11 Fig — (PDF) [file pgen.1011545.s020.pdf]

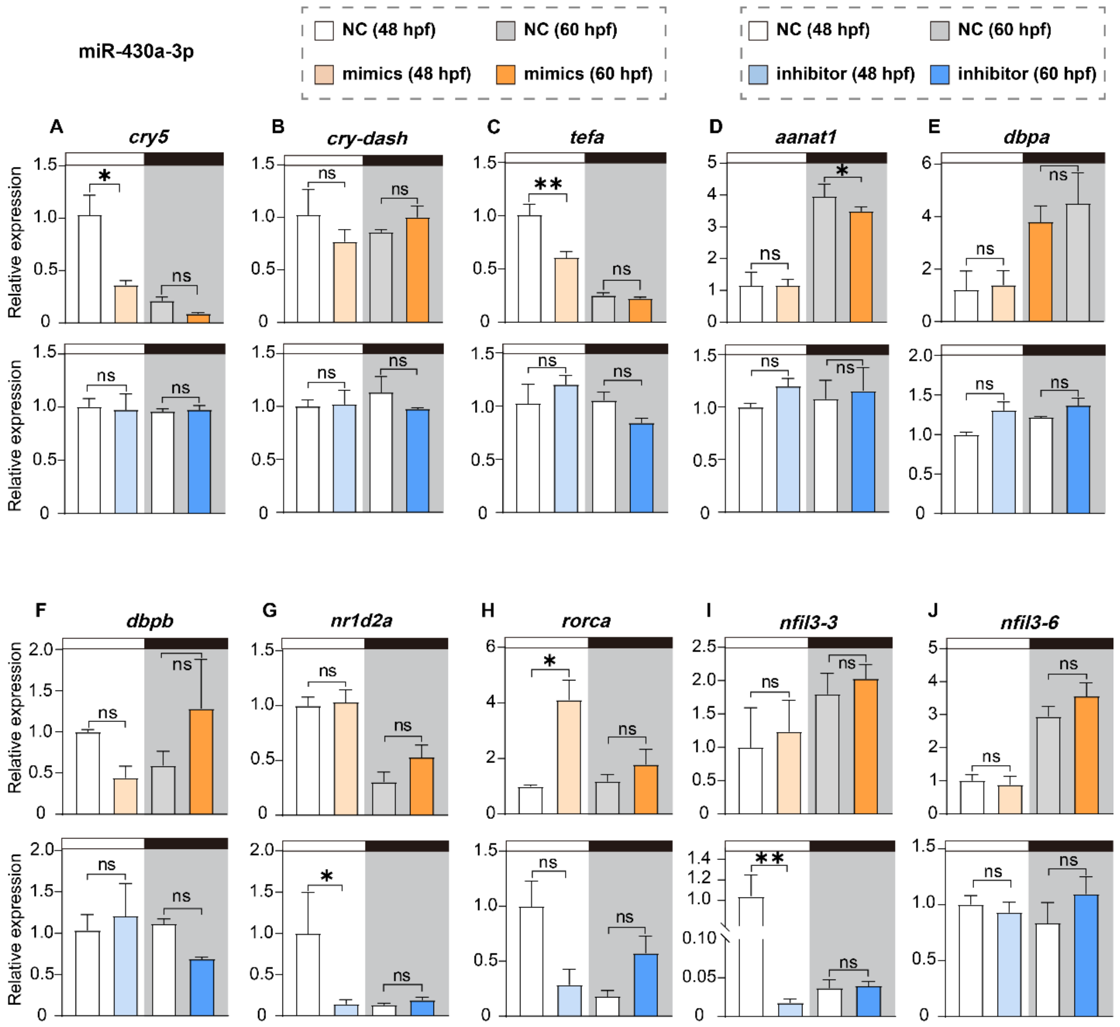

S11 Fig. The effects of miR-430a-3p on the other circadian clock and DNA repair genes. The values are presented as mean ± SEM in histograms. One-way ANOVA or Kruskal-Wallis test followed by multiple comparisons test results are reported in **S5 Table**. Significant differences are indicated by asterisks (***$p < 0.001$, **$p < 0.01$, *$p < 0.05$).
