# Supplementary material for: Light-regulated microRNAs shape dynamic gene expression in the zebrafish circadian clock
Source: PLoS Genet. 2025 Jan 8;21(1):e1011545. doi: 10.1371/journal.pgen.1011545 (PMC11750094; doi:10.1371/journal.pgen.1011545)
Supplement: S12 Fig — (PDF) [file pgen.1011545.s021.pdf]

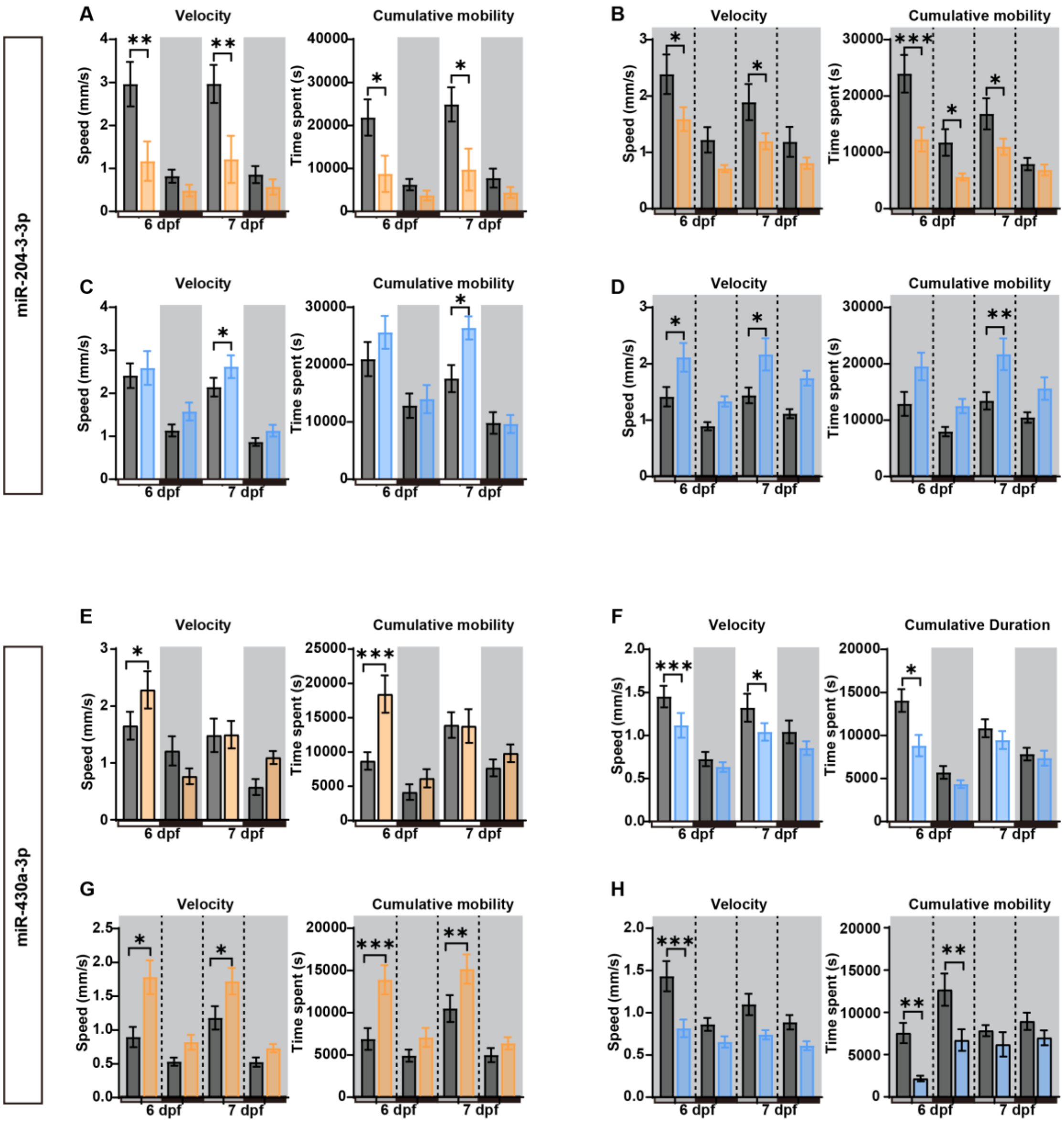

S12 Fig. Light-responsive miRNAs modulate the rhythmic activity of zebrafish larvae. (A-D) Rhythmic locomotor activity of zebrafish larvae upon miR-204-3-3p overexpression or suppression during LD cycle or DD condition. **(E-H)** Rhythmic locomotor activity of zebrafish larvae upon miR-430a-3p overexpression or suppression during LD cycle or DD condition. The values are presented as mean ± SEM in histograms. Paired t-test was performed and the results are reported in **S4 Table**. Significant differences are indicated by asterisks (***$p < 0.001$, **$p < 0.01$, *$p < 0.05$).
